# Supplementary material for: Increased levels of eIF2A inhibit translation by sequestering 40S ribosomal subunits
Source: Nucleic Acids Res. 2023 Aug 21;51(18):9983–10000. doi: 10.1093/nar/gkad683 (PMC10570035; doi:10.1093/nar/gkad683)
Supplement: gkad683_Supplemental_File [file gkad683_supplemental_file.pdf]

SUPPLEMENTARY DATA – Grove et al.

Supplementary Table S1. Values for heat map in Supplementary Figure S1.

Cell Line	Tissue/Cell Type	eIF2α (ppm)	eIF2β (ppm)	eIF2γ (ppm)	eIF2A (ppm)
HeLa	Cervical	213	204	110	198
A549	Lung	214	196	109	106
HEK293	Kidney	182	178	92.5	167
MCF7	Breast	206	191	102	190
U2OS	Bone	204	181	96.7	229
Jurkat	T lymphocyte	217	213	109	250
LnCap	Prostate	202	194	102	185
RKO	Colon	233	208	117	210
HepG2	Liver	226	213	114	202

Supplementary Table S2. Oligonucleotides used in this study.

Oligo Name	Sequence (5'-3')
F_nLuc qPCR	CAGCCGGCTACAACCTGGAC
R_nLuc qPCR	AGCCCATTTTCACCGCTCAG
F_control FFLuc qPCR	AACGCTTCCATCTTCCAGGG
R_control FFLuc qPCR	CCAGATCCACAACCTTCGCT

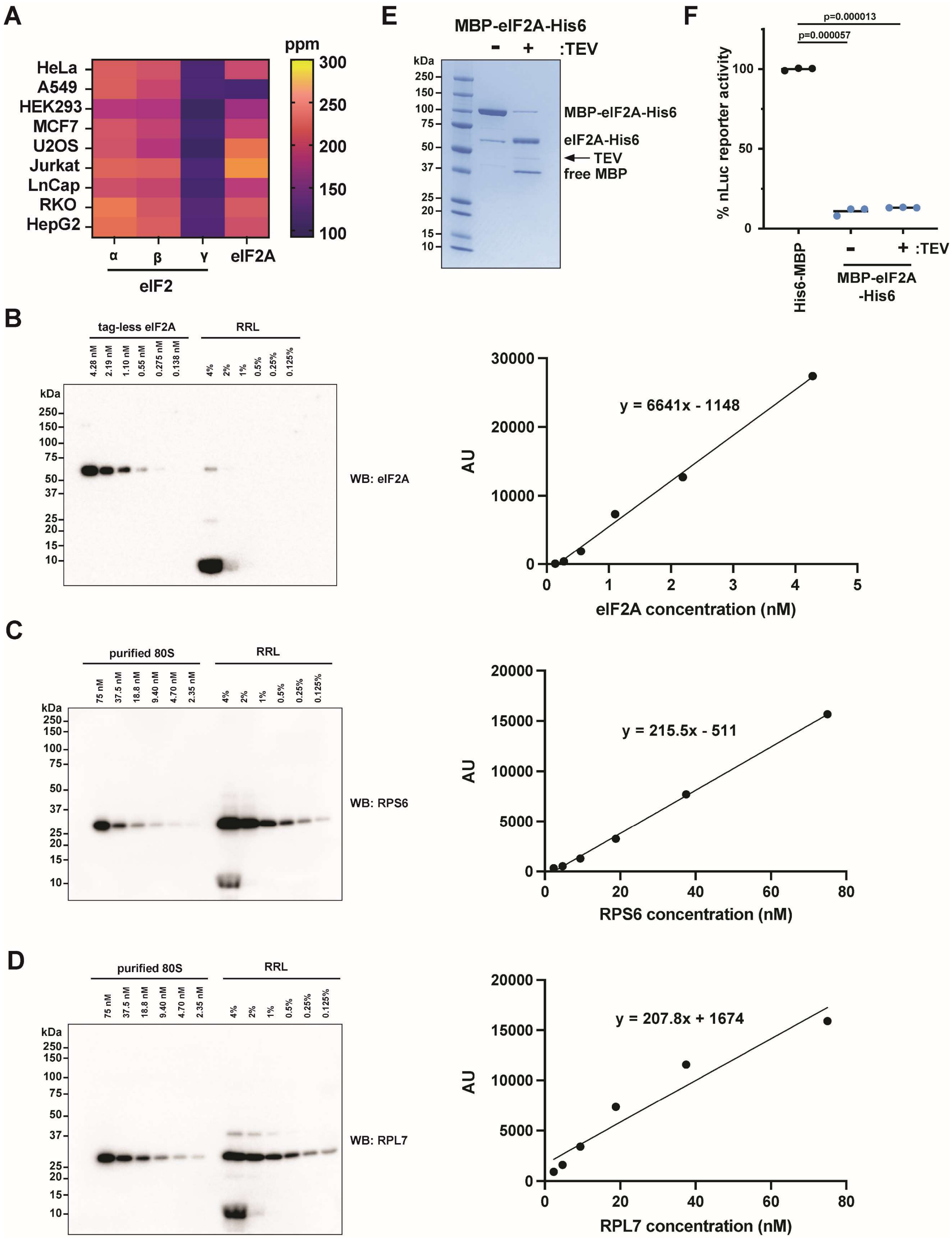

Supplementary Figure S1. eIF2(α,β,γ) and eIF2A are expressed at similar levels in various human cell types and eIF2A-mediated inhibition remains with TEV protease cleavage. A) Comparison of endogenous eIF2(α, β, γ) and eIF2A protein levels in various human cell types.

Data was obtained from pax-db.org using the Geiger, MCP, 2012 dataset. Ppm = parts per million. It should be noted that pax-db.org also contains data sets calculated from spectral counts (these data sets have "SC" in their name) that are less confident due to the lower protein coverage. B-D) Calculation of eIF2A (B), RPS6 (C), and RPL7 (D) concentrations in RRL by Western blot. Recombinant tag-less eIF2A and purified 80S ribosomes from RRL were titrated as a standard curve; quantification showed signal was in the linear dynamic range. The intensity of the 4% RRL sample was used for eIF2A and the 1% RRL sample was used for RPS6 and RPL7 in the line equation and then multiplied by a dilution factor of 5 or 20, respectively, to determine the concentration of each protein in 20% RRL. For RPS6 and RPL7, these concentrations were averaged since 40S and 60S ribosomal subunits are equimolar. E) SDS-PAGE and Coomassie stain of MBP-eIF2A-His6 without and with TEV protease treatment. F) Response of *in vitro* translation reactions programmed with nLuc mRNA supplemented with 1.68 μM mock-cleaved and 1.68 μM TEV protease-cleaved MBP-eIF2A-His6. Bars represent the mean. n=3 biological replicates. Comparisons were made using a two-tailed unpaired t-test with Welch's correction.

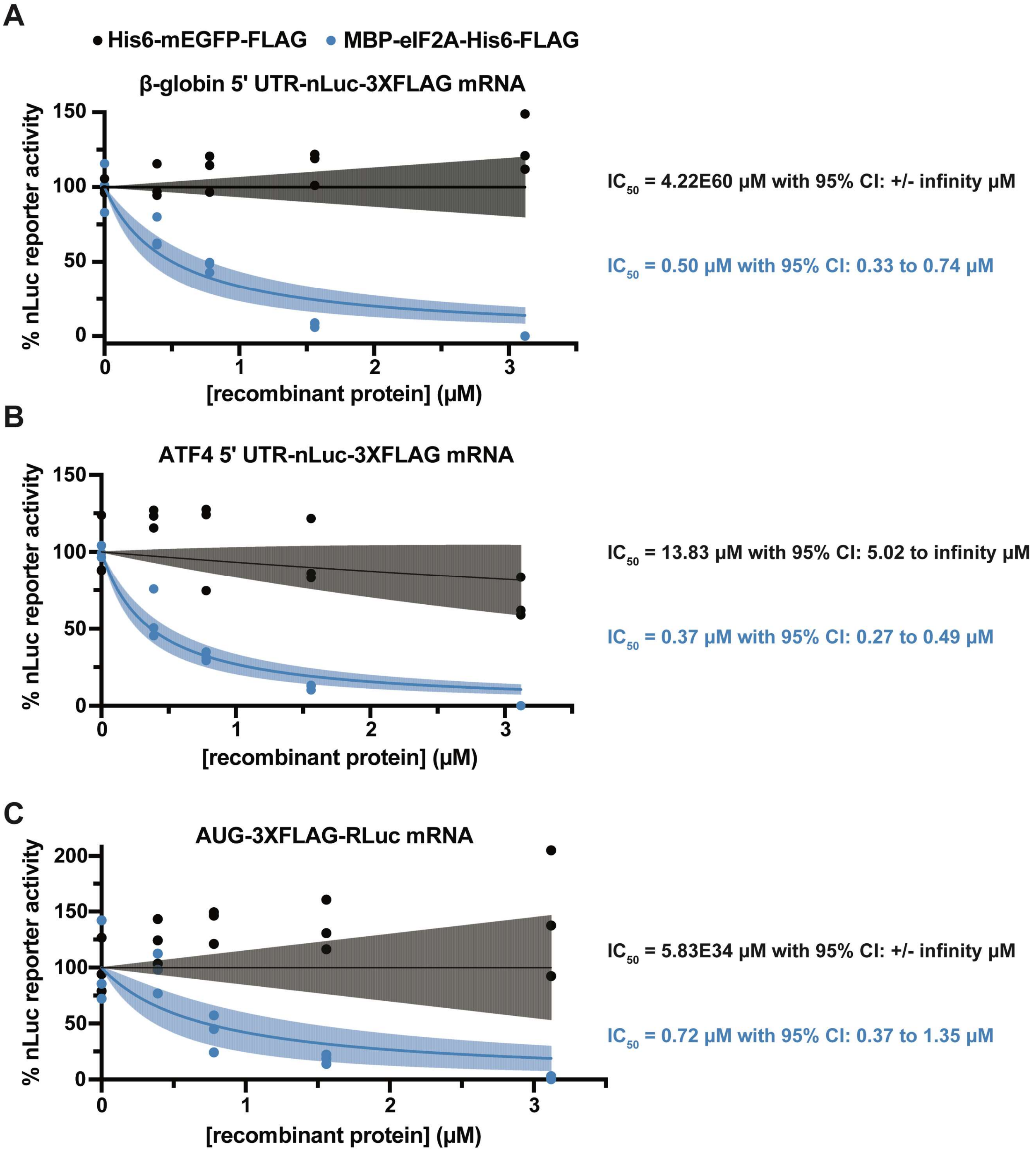

Supplementary Figure S2. Recombinant eIF2A inhibits translation of mRNAs with different 5' UTRs and coding sequences. A-C) *In vitro* translation of different reporter mRNAs with a titration (0-3.12 μM) of His6-mEGFP-FLAG or MBP-eIF2A-His6-FLAG. mRNAs tested were β-globin 5' UTR nLuc mRNA (A), ATF4 5' UTR nLuc mRNA (B), and AUG-3XFLAG-RLuc mRNA (C). n=3 biological replicates. A non-linear regression was used to calculate the IC_{50} and is shown as the line with the 95% CI included as a watermark.

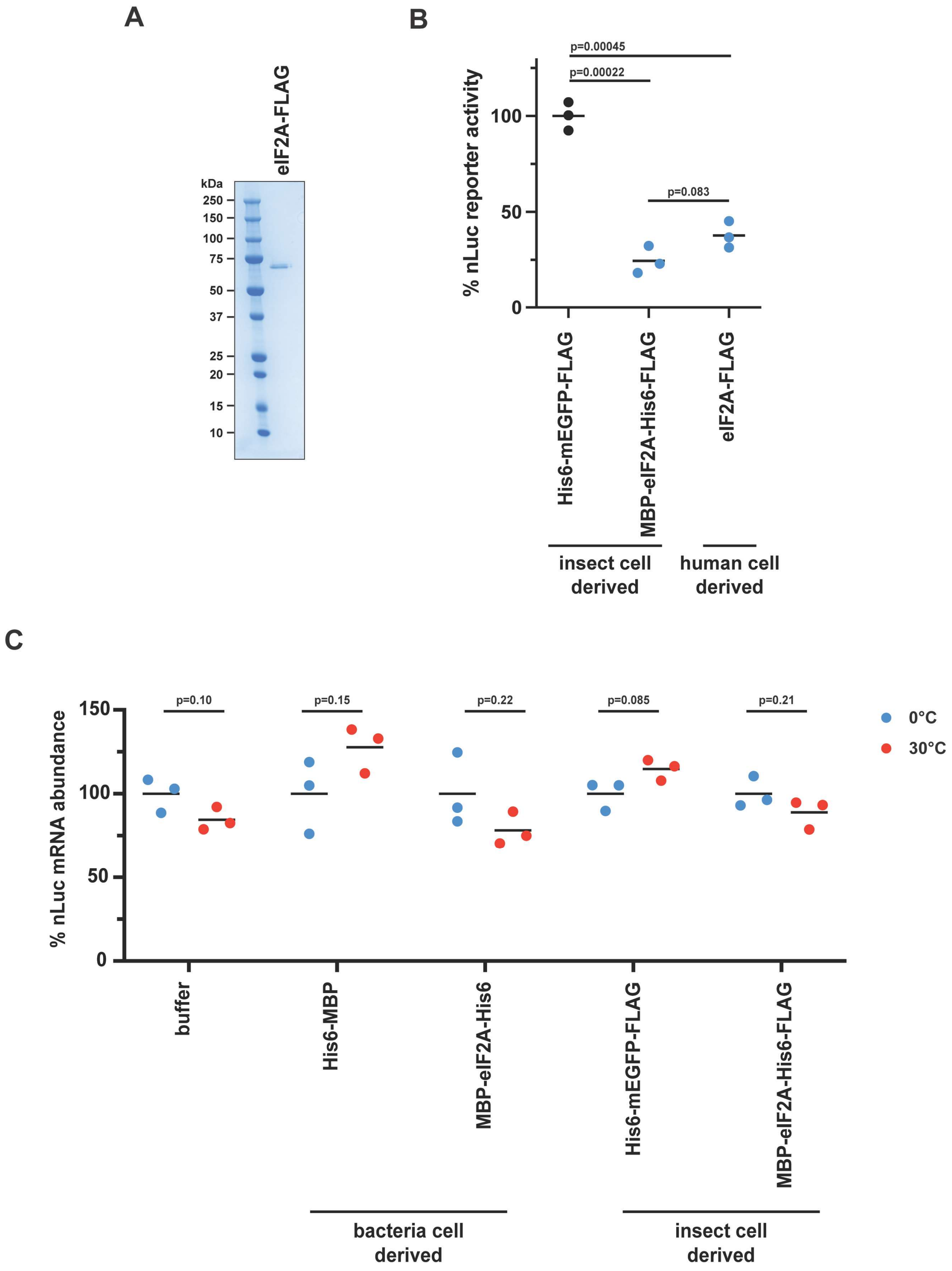

Supplementary Figure S3. Human cell derived eIF2A-FLAG is inhibitory and recombinant eIF2A does not alter reporter mRNA levels during translation. A) SDS-PAGE and Coomassie stain of recombinant eIF2A-FLAG expressed and purified from HEK293T cells (obtained from OriGene). 1 µg was loaded. B) Response of *in vitro* translation reactions

programmed with nLuc reporter mRNAs in the presence of 1.2 μM insect cell derived His6-mEGFP-FLAG, 1.2 μM insect cell derived MBP-eIF2A-His6-FLAG, or 1.2 μM human cell derived eIF2A-FLAG. Bars represent the mean. n=3 biological replicates. Comparisons were made using a two-tailed unpaired t-test with Welch's correction. C) Relative levels of nLuc reporter mRNA before and after translation with Protein Storage Buffer, 1.68 μM *E. coli* derived His6-MBP, 1.68 μM *E. coli* derived MBP-eIF2A-His6, 1.68 μM insect cell derived His6-mEGFP-FLAG, or 1.68 μM insect cell derived MBP-eIF2A-His6-FLAG. Separate identical reactions were either left on ice (0°C) or translated at 30°C for 30 min. nLuc mRNA abundance for each condition was relative to 0°C samples kept on ice. Reactions were spiked with 0.2 ng control FFLuc mRNA before total RNA was extracted using TRIzol. cDNA was subsequently synthesized and analyzed by RT-qPCR. Bars represent the mean. n=3 biological replicates. Comparisons were made using a two-tailed unpaired t-test with Welch's correction.

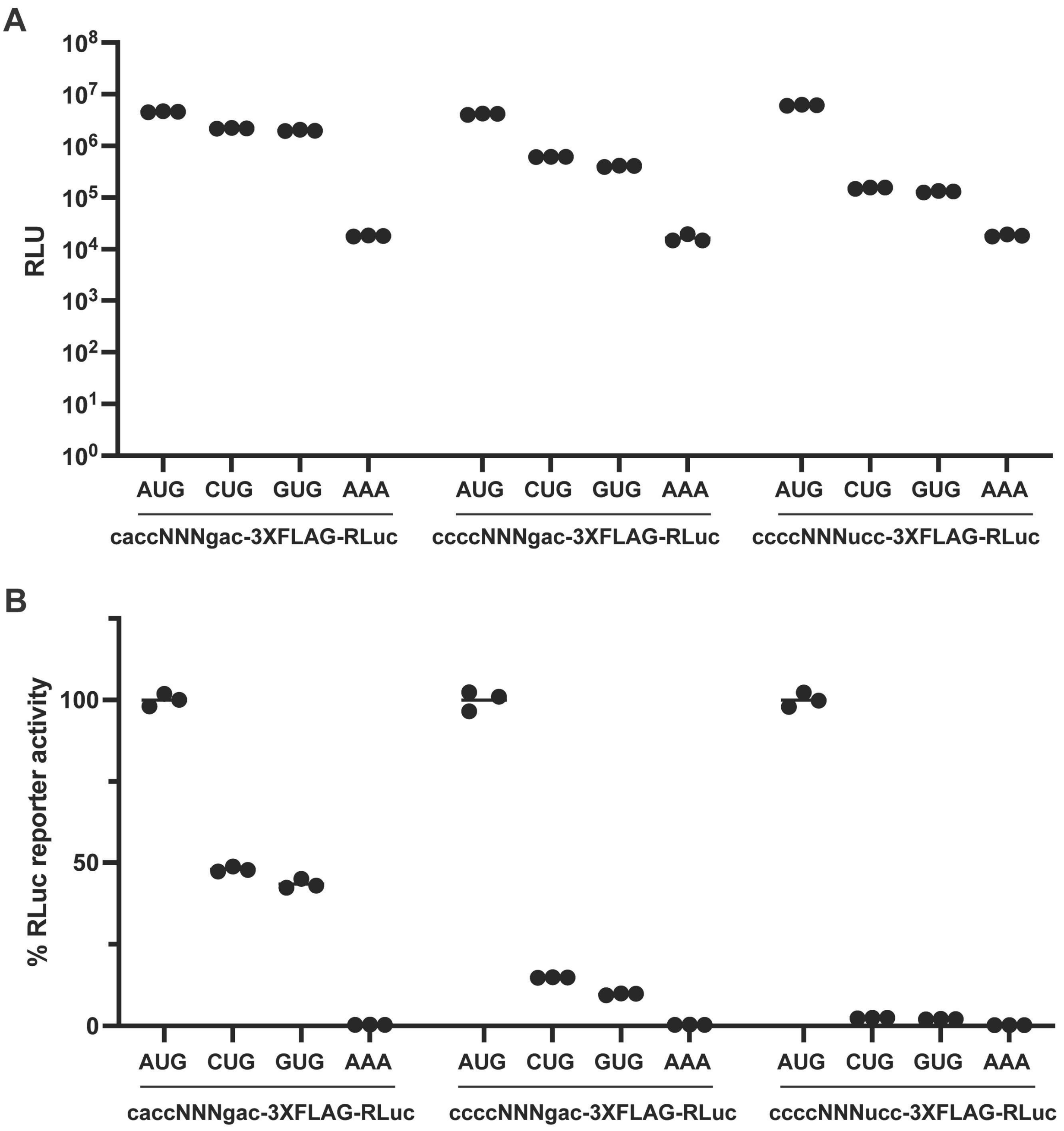

Supplementary Figure S4. Optimization of RLuc reporter mRNAs. A) *In vitro* translation of nLuc reporter mRNA set 1 (caccNNNguc-3XFLAG-RLuc), set 2 (ccccNNNguc-3XFLAG-RLuc), or set 3 (ccccNNNucc-3XFLAG-RLuc) in RRL. The Kozak sequence surrounding the AUG or non-AUG start codon is perfect (set 1) or mutated (sets 2 and 3). Wei *et al*. has shown that imperfect Kozak sequences cause more drastic efficiencies between AUG and near-cognate start codons in RRL (49). Raw luciferase values (relative luciferase units; RLU) are reported. Bars represent the mean. n=3 biological replicates. B) Same as in A, but each reporter set is relative to the respective AUG-encoded reporter. Bars represent the mean. n=3 biological replicates. Set 3 reporters are used in **Figure 2** and **Supplementary Figure S5**. The AUG-RLuc in set 1 is used in **Supplementary Figure S2C**. Complete sequences of these reporters are provided in the **Supplementary Data**.

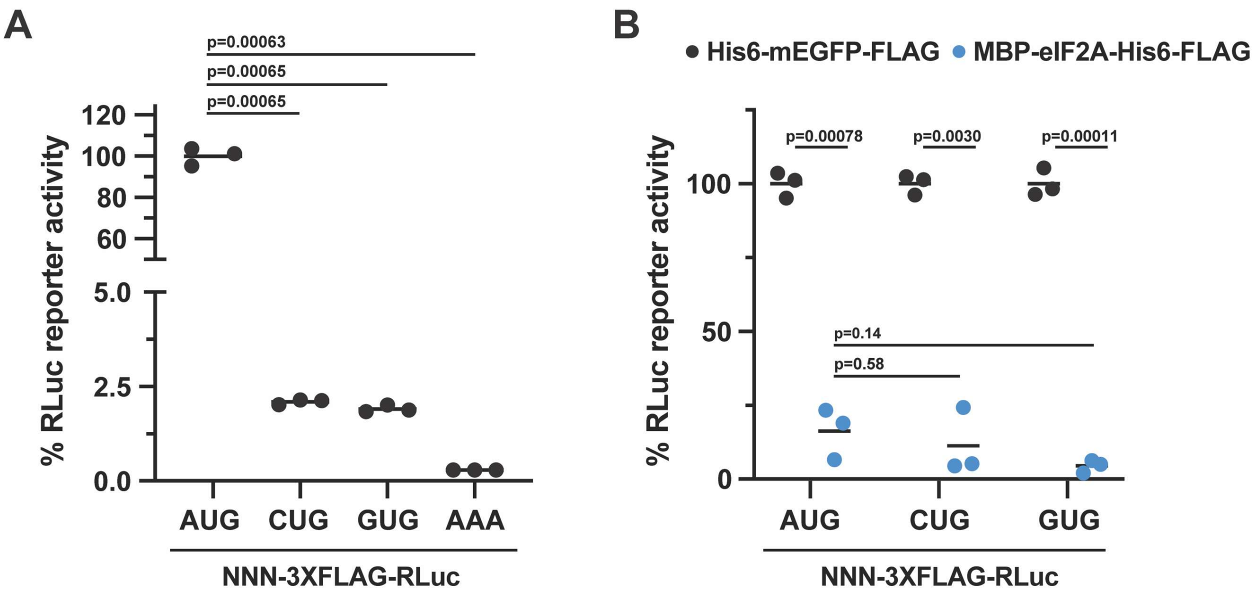

Supplementary Figure S5. Insect cell derived eIF2A inhibits AUG- and non-AUG translation. A) Comparison of AUG- and non-AUG-3XFLAG-RLuc reporter mRNAs translated *in vitro* in the presence of 1.68 μM His6-mEGFP-FLAG. Set 3 RLuc reporters from **Supplementary Figure S4** were used. Luciferase levels are normalized to AUG-3XFLAG-RLuc. Bars represent the mean. n=3 biological replicates. Comparisons were made using a two-tailed unpaired t-test with Welch's correction. B) Response of *in vitro* translation reactions programmed with AUG- and non-AUG-3XFLAG-RLuc reporter mRNAs in the presence of 1.68 μM His6-mEGFP-FLAG or 1.68 μM MBP-eIF2A-His6-FLAG. Bars represent the mean. n=3 biological replicates. Comparisons were made using a two-tailed unpaired t-test with Welch's correction.

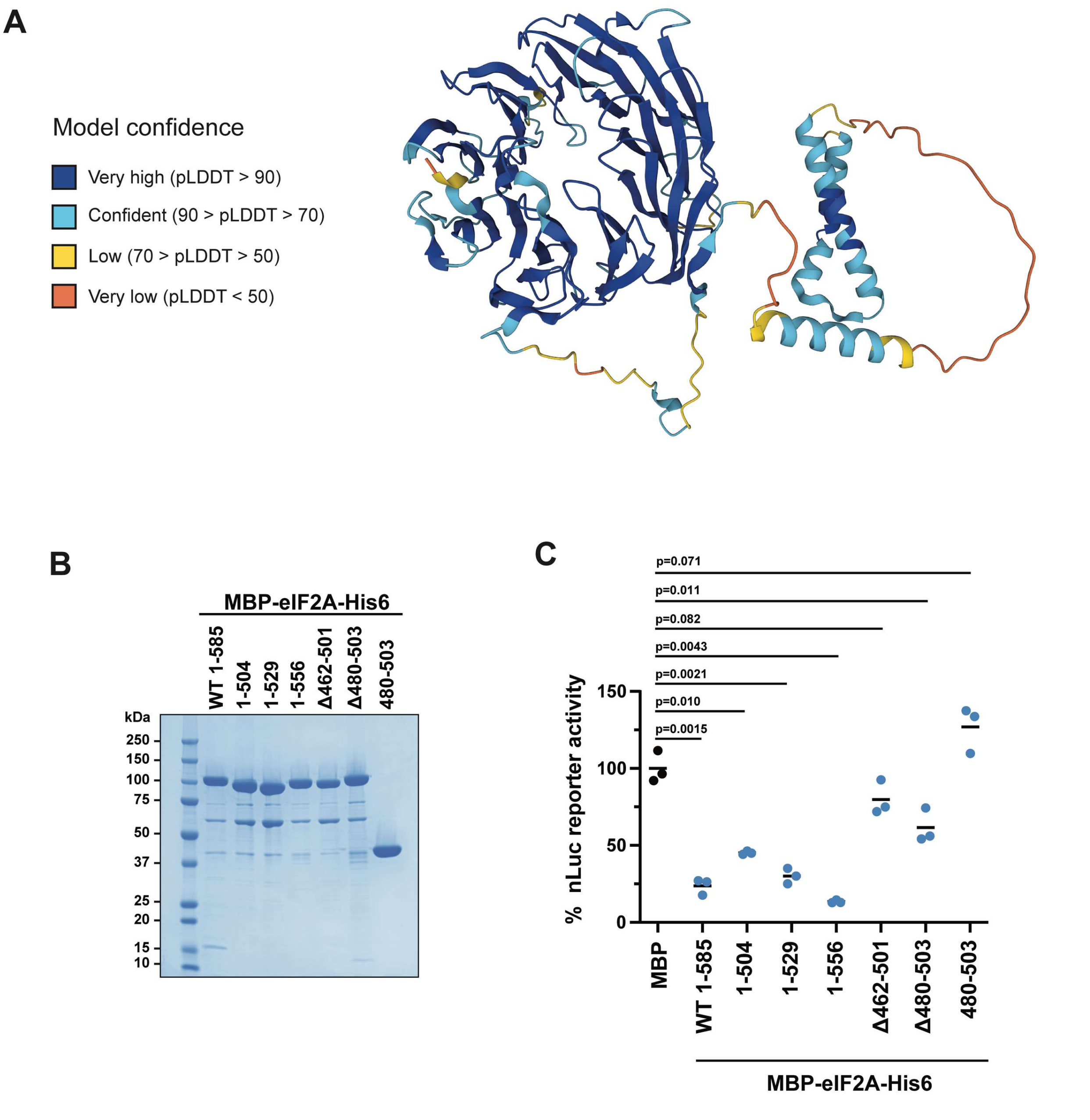

Supplementary Figure S6. Single domain mutants of eIF2A are not translationally repressive. A) AlphaFold structural prediction of full-length human eIF2A with confidence coloring, which uses the predicted local distance difference test (pLDDT). B) SDS-PAGE and Coomassie stain of recombinant MBP-eIF2A-His6 mutants. 2 μg of protein was loaded. C) Response of *in vitro* translation reactions programmed with nLuc mRNA in the presence of 1.68 μM of the indicated recombinant protein. Bars represent the mean. n=3 biological replicates. Comparisons were made using a two-tailed unpaired t-test with Welch’s correction.

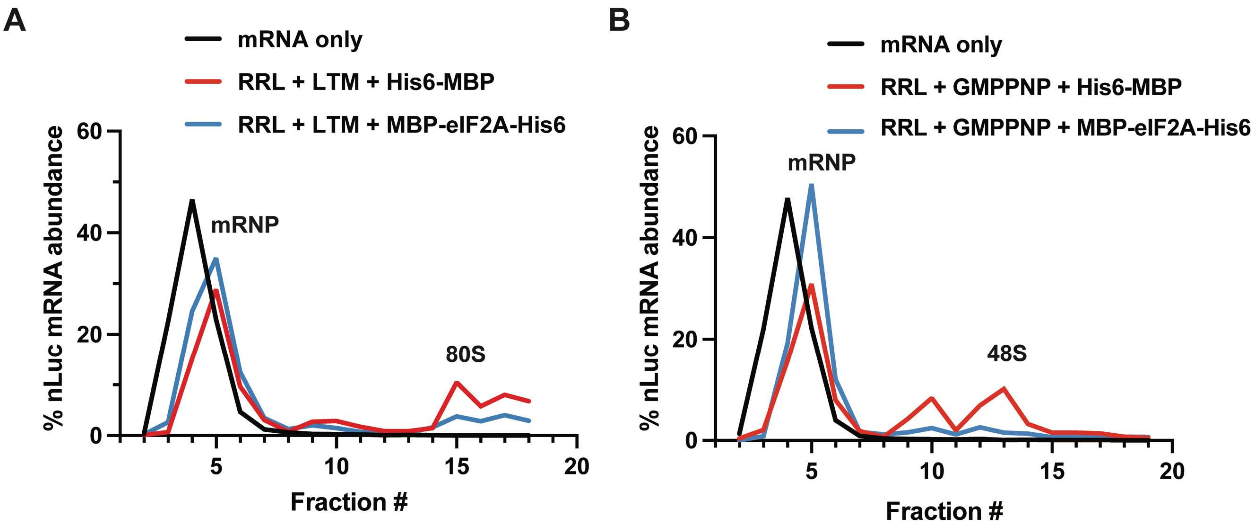

Supplementary Figure S7. eIF2A represses translation initiation before 48S initiation complex formation. Replicate gradients as shown in **Figure 4**. A) nLuc mRNA distribution along a 5-30% (w/v) buffered sucrose gradient. *In vitro* translation reactions were supplemented with 50 μM lactimidomycin (LTM) to stall 80S ribosomes before the first translocation cycle and with either 1.68 μM His6-MBP or 1.68 μM MBP-eIF2A-His6, then diluted and separated on buffered sucrose gradients. B) Same as in A, but instead supplemented with 5 mM GMPPNP to capture 48S initiation complexes at the start codon.

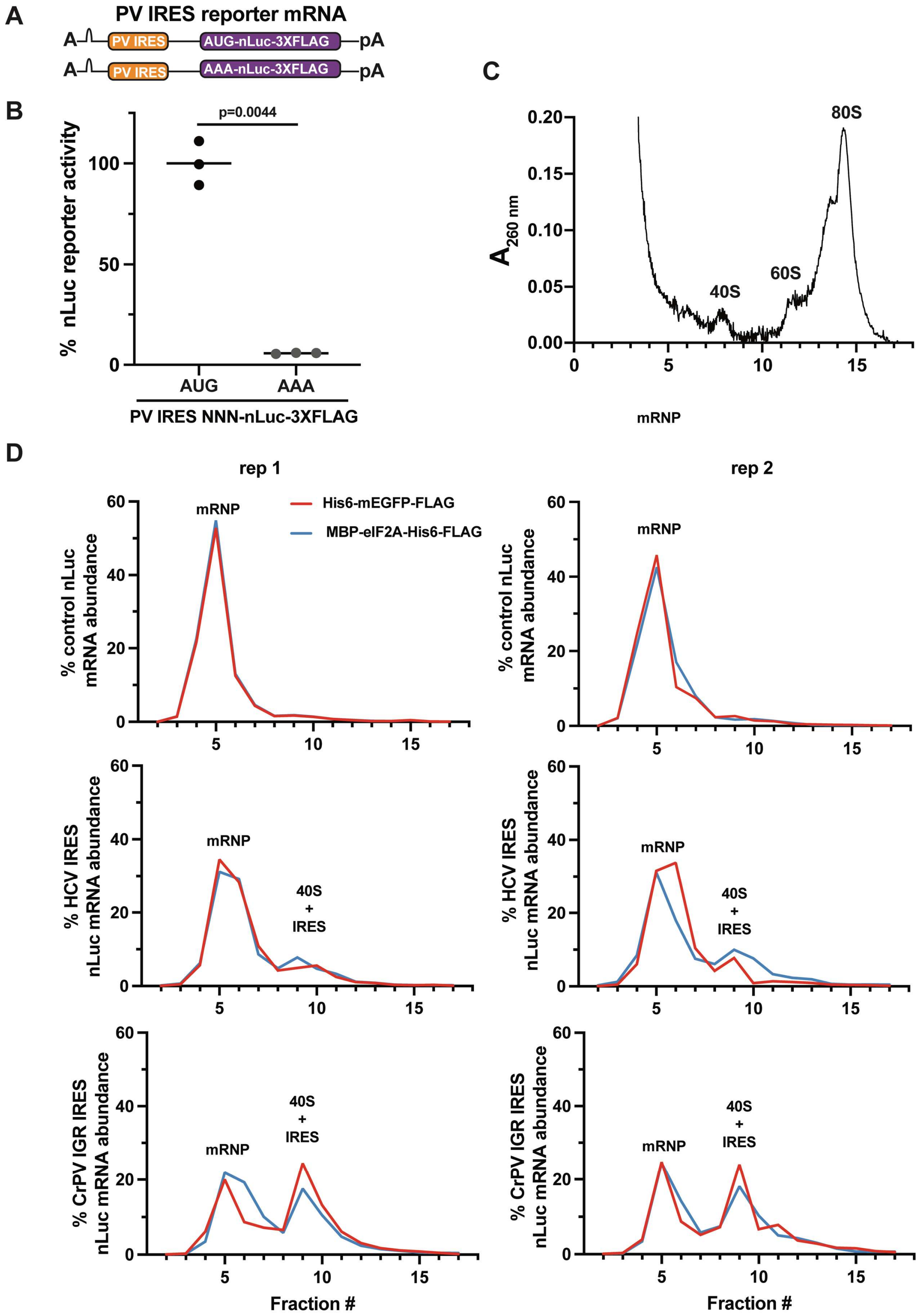

Supplementary Figure S8. Control experiments with PV, HCV, and CrPV IGR IRES nLuc mRNA reporters. A) Schematic of PV IRES nLuc reporters with the nLuc ORF harboring an AUG or AAA start codon. B) Comparison of *in vitro* translation reactions programmed with either

PV IRES nLuc mRNA or PV IRES (AUG to AAA) nLuc mRNA. The AUG to AAA mutation dramatically reduced the signal produced from the reporter, providing evidence that the desired AUG start codon of nLuc is primarily being used in RRL. Bars represent the mean. n=3 biological replicates. Comparisons were made using a two-tailed unpaired t-test with Welch's correction. C-D) The ability of nLuc mRNA, HCV IRES nLuc mRNA, and CrPV IGR IRES nLuc mRNA to interact with 40S ribosomal subunits and 80S ribosomes in RRL was assessed by 5-30% (w/v) sucrose gradient ultracentrifugation. Translation reactions were assembled identically as in **Figure 5B** with control (no IRES) nLuc mRNA, HCV IRES nLuc mRNA, or CrPV IGR IRES nLuc mRNA in the presence of 1.68 µM His6-mEGFP-FLAG or 1.68 µM MBP-eIF2A-His6-FLAG but incubated on ice for 30 min to allow interactions between the mRNAs and translation machinery (samples were *NOT* incubated at 30°C as in **Figure 5B**). Reactions were then diluted and separated on buffered sucrose gradients, and reporter mRNA abundance was measured across the gradient as described in the Materials and Methods. Duplicates are shown side-by-side. The $A_{260\ nm}$ trace of an untranslated RRL reaction (assembled and kept on ice, *NOT* incubated at 30°C) is shown in C. Control (no IRES) nLuc mRNA did not co-sediment with 40S subunits. A significant proportion of the HCV IRES nLuc mRNA and CrPV IGR IRES nLuc mRNA did sediment in 40S-containing fractions, with a higher proportion of CrPV IGR IRES nLuc mRNA being found in the 40S-containing fractions than HCV IRES nLuc mRNA. These data demonstrate that these assay conditions are favorable for both IRESs to stably interact with the 40S subunit. Despite the CrPV IGR IRES nLuc mRNA being able to bind pre-formed vacant 80S ribosomes from salt-washed 40S and 60S subunits (64), little to no CrPV IGR IRES nLuc mRNA sedimented in the 80S-containing fractions. Interestingly, although inhibiting translation of the HCV IRES and CrPV IGR IRES nLuc reporter mRNAs (**Figure 5B**), addition of recombinant eIF2A did not robustly prevent both IRES nLuc mRNAs from co-sedimenting with 40S subunits. Nor did eIF2A cause more IRES nLuc mRNAs to accumulate at the top of the gradient as would be expected if eIF2A was interfering with 40S subunits interacting with either IRES. To speculate, these data overall may suggest that eIF2A is preventing mRNA from being stably inserted into the mRNA channel in the 40S subunit. Unpublished eCLIP data for eIF2A from Wei *et al*. provides some evidence that eIF2A crosslinks to rRNA in the vicinity of the mRNA exit channel (87). The positioning/structure of eIF2A on the 40S is not empirically determined in Wei *et al.* when our manuscript was in preparation or in any other manuscript that we know of. Even for IRESs, which bind to the E, P, and/or A sites of the ribosome first, the mRNA must still be inserted into the mRNA channel after initiation to efficiently allow the ribosome to decode and translocate along the coding sequence. In canonical cap- and scanning-dependent translation, eIF4F bound to the mRNA recruits the 43S PIC and the mRNA is then loaded into the mRNA channel of the 40S subunit; however, this complex is not stable like IRES•40S complexes until start codon recognition occurs and the canonical 48S initiation complex is formed.

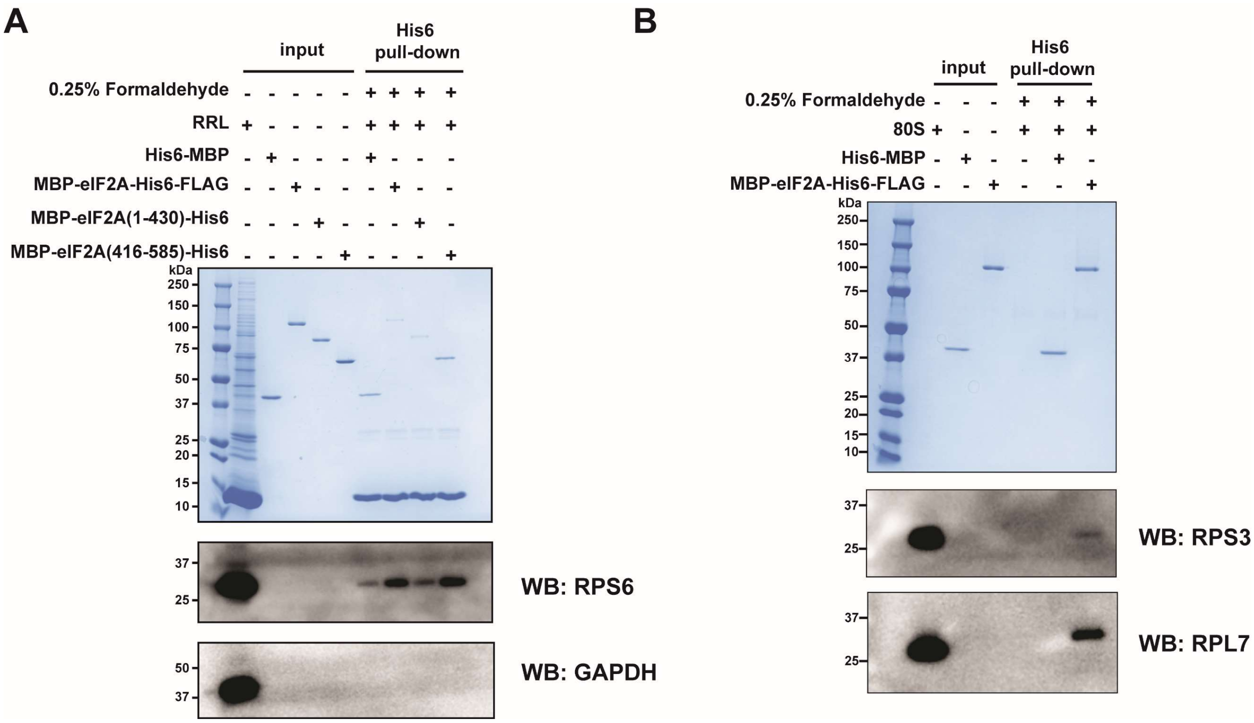

Supplementary Figure S9. Control pulldown experiments with deletion mutants and 80S ribosomes. A) SDS-PAGE, Coomassie stain, and Western blot analysis to assess the ability of recombinant His6-MBP and the indicated recombinant His6-tagged eIF2A proteins to pulldown 40S subunits from RRL with Ni^{2+}-NTA magnetic beads. 0.25% (v/v) formaldehyde was included in the indicated samples. RPS6 was used as a marker for 40S subunits. GAPDH was used as a negative control. Coomassie staining was used to confirm capture of His6-tagged recombinant proteins. Unequal pulldown was noted between full-length and deletion mutants in these cross-linking conditions. B) SDS-PAGE, Coomassie stain, and Western blot analysis to assess the ability of recombinant His6-MBP or MBP-eIF2A-His6-FLAG to pulldown purified 80S ribosomes with Ni^{2+}-NTA magnetic beads. 0.25% (v/v) formaldehyde was included in the indicated samples. RPS3 and RPL7 were used as markers for 40S and 60S subunits, respectively. Coomassie staining was used to confirm capture of His6-tagged recombinant proteins.

Reporter mRNA sequences
T7 promoter
start codon
3XFLAG
nLuc
RLuc
stop codon
Hairpin
IRES

nLuc-3XFLAG [control (no IRES) nLuc]
TAATACGACTCACTATAGGAGACCCAAGCTGGCTAGCGTTTAAACTTAAGCTTGGCAATCC
GGTACTGTTGGTAAATAAGCCACCATGGTCTTCACACTCGAAGATTTCGTTGGGGACTGGC
GACAGACAGCCGGCTACAACCTGGACCAAGTCCTTGAACAGGGAGGTGTGTCCAGTTTGT
TTCAGAATCTCGGGGTGTCCGTAACTCCGATCCAAAGGATTGTCCTGAGCGGTGAAAATG
GGCTGAAGATCGACATCCATGTCATCATCCCGTATGAAGGTCTGAGCGGCGACCAAATGG
GCCAGATCGAAAAAATTTTTAAGGTGGTGTACCCTGTGGATGATCATCACTTTAAGGTGATC
CTGCACTATGGCACACTGGTAATCGACGGGGTTACGCCGAACATGATCGACTATTTCGGA
CGGCCGTATGAAGGCATCGCCGTGTTCGACGGCAAAAAGATCACTGTAACAGGGACCCTG
TGGAACGGCAACAAAATTATCGACGAGCGCCTGATCAACCCCGACGGCTCCCTGCTGTTC
CGAGTAACCATCAACGGAGTGACCGGCTGGCGGCTGTGCGAACGCATTCTGGCGGACTA
CAAAGACCATGACGGTGATTATAAAGATCATGACATCGATTACAAGGATGACGATGACAAG
TAAGGCCGCGACTCTAGAGGGCCC

β-globin 5' UTR-nLuc-3XFLAG
TAATACGACTCACTATAGGGACATTTGCTTCTGACACAACTGTGTTCACTAGCAACCTCAAA
CAGACACCATGGTCTTCACACTCGAAGATTTCGTTGGGGACTGGCGACAGACAGCCGGCT
ACAACCTGGACCAAGTCCTTGAACAGGGAGGTGTGTCCAGTTTGTTTCAGAATCTCGGGGT
GTCCGTAACTCCGATCCAAAGGATTGTCCTGAGCGGTGAAAATGGGCTGAAGATCGACAT
CCATGTCATCATCCCGTATGAAGGTCTGAGCGGCGACCAAATGGGCCAGATCGAAAAAATT
TTTAAGGTGGTGTACCCTGTGGATGATCATCACTTTAAGGTGATCCTGCACTATGGCACAC
TGGTAATCGACGGGGTTACGCCGAACATGATCGACTATTTCGGACGGCCGTATGAAGGCA
TCGCCGTGTTCGACGGCAAAAAGATCACTGTAACAGGGACCCTGTGGAACGGCAACAAAA
TTATCGACGAGCGCCTGATCAACCCCGACGGCTCCCTGCTGTTCCGAGTAACCATCAACG
GAGTGACCGGCTGGCGGCTGTGCGAACGCATTCTGGCGGACTACAAAGACCATGACGGT
GATTATAAAGATCATGACATCGATTACAAGGATGACGATGACAAGTAAGGCCGCGACTCTA
GAGGGCCC

ATF4 5' UTR-nLuc-3XFLAG
TAATACGACTCACTATAGGGATCCTTTCTACTTTGCCCGCCCACAGATGTAGTTTTCTCTGC
GCGTGTGCGTTTCCCTCCTCCCCGCCCTCAGGGTCCACGGCCACCATGGCGTATTAGGG
GCAGCAGTGCCTGCGGCAGCATTGGCCTTTGCAGCGGCGGCAGCAGCACCAGGCTCTGC
AGCGGCAACCCCCAGCGGCTTAAGCCATGGCGCTTCTCACGGCATTCAGCAGCAGCGTTG
CTGTAACCGACAAAGACACCTTCGAATTAAGCACATTCCTCGATTCCAGCAAAGCACCGCA
ACATGGTCTTCACACTCGAAGATTTCGTTGGGGACTGGCGACAGACAGCCGGCTACAACC
TGGACCAAGTCCTTGAACAGGGAGGTGTGTCCAGTTTGTTTCAGAATCTCGGGGTGTCCG
TAACTCCGATCCAAAGGATTGTCCTGAGCGGTGAAAATGGGCTGAAGATCGACATCCATGT
CATCATCCCGTATGAAGGTCTGAGCGGCGACCAAATGGGCCAGATCGAAAAAATTTTTAAG
GTGGTGTACCCTGTGGATGATCATCACTTTAAGGTGATCCTGCACTATGGCACACTGGTAA
TCGACGGGGTTACGCCGAACATGATCGACTATTTCGGACGGCCGTATGAAGGCATCGCCG
TGTTCGACGGCAAAAAGATCACTGTAACAGGGACCCTGTGGAACGGCAACAAAATTATCGA

CGAGCGCCTGATCAACCCCGACGGCTCCCTGCTGTTCCGAGTAACCATCAACGGAGTGAC
CGGCTGGCGGCTGTGCGAACGCATTCTGGCGGACTACAAAGACCATGACGGTGATTATAA
AGATCATGACATCGATTACAAGGATGACGATGACAAGTAAGGCCGCGACTCTAGAGGGCC
C

AUG-3XFLAG-RLuc (set 1)
TAATACGACTCACTATAGGGAGACCCAAGCTGGCTAGCGTTTAAACTTAAGCTTGGCAATC
CGGTACTGTTGGTAAATAAGCCACCATGGACTACAAAGACCATGACGGTGATTATAAAGAT
CATGACATCGATTACAAGGATGACGATGACAAGACTTCGAAAGTTTATGATCCAGAACAAA
GGAAACGGATGATAACTGGTCCGCAGTGGTGGGCCAGATGTAAACAAATGAATGTTCTTGA
TTCATTTATTAATTATTATGATTCAGAAAAACATGCAGAAAATGCTGTTATTTTTTTACATGGT
AACGCGGCCTCTTCTTATTTATGGCGACATGTTGTGCCACATATTGAGCCAGTAGCGCGGT
GTATTATACCAGACCTTATTGGTATGGGCAAATCAGGCAAATCTGGTAATGGTTCTTATAGG
TTACTTGATCATTACAAATATCTTACTGCATGGTTTGAACTTCTTAATTTACCAAAGAAGATC
ATTTTTGTCGGCCATGATTGGGGTGCTTGTTTGGCATTTCATTATAGCTATGAGCATCAAGA
TAAGATCAAAGCAATAGTTCACGCTGAAAGTGTAGTAGATGTGATTGAATCATGGGATGAAT
GGCCTGATATTGAAGAAGATATTGCGTTGATCAAATCTGAAGAAGGAGAAAAAATGGTTTT
GGAGAATAACTTCTTCGTGGAAACCATGTTGCCATCAAAAATCATGAGAAAGTTAGAACCA
GAAGAATTTGCAGCATATCTTGAACCATTCAAAGAGAAAGGTGAAGTTCGTCGTCCAACATT
ATCATGGCCTCGTGAAATCCCGTTAGTAAAAGGTGGTAAACCTGACGTTGTACAAATTGTTA
GGAATTATAATGCTTATCTACGTGCAAGTGATGATTTACCAAAAATGTTTATTGAATCGGAC
CCAGGATTCTTTTCCAATGCTATTGTTGAAGGTGCCAAGAAGTTTCCTAATACTGAATTTGT
CAAAGTAAAAGGTCTTCATTTTTCGCAAGAAGATGCACCTGATGAAATGGGAAAATATATCA
AATCGTTCGTTGAGCGAGTTCTCAAAAATGAACAATAAGGCCGCGACTCTAGAGGGCCC

CUG-3XFLAG-RLuc (set 1)
TAATACGACTCACTATAGGGAGACCCAAGCTGGCTAGCGTTTAAACTTAAGCTTGGCAATC
CGGTACTGTTGGTAAATAAGCCACCCTGGACTACAAAGACCATGACGGTGATTATAAAGAT
CATGACATCGATTACAAGGATGACGATGACAAGACTTCGAAAGTTTATGATCCAGAACAAA
GGAAACGGATGATAACTGGTCCGCAGTGGTGGGCCAGATGTAAACAAATGAATGTTCTTGA
TTCATTTATTAATTATTATGATTCAGAAAAACATGCAGAAAATGCTGTTATTTTTTTACATGGT
AACGCGGCCTCTTCTTATTTATGGCGACATGTTGTGCCACATATTGAGCCAGTAGCGCGGT
GTATTATACCAGACCTTATTGGTATGGGCAAATCAGGCAAATCTGGTAATGGTTCTTATAGG
TTACTTGATCATTACAAATATCTTACTGCATGGTTTGAACTTCTTAATTTACCAAAGAAGATC
ATTTTTGTCGGCCATGATTGGGGTGCTTGTTTGGCATTTCATTATAGCTATGAGCATCAAGA
TAAGATCAAAGCAATAGTTCACGCTGAAAGTGTAGTAGATGTGATTGAATCATGGGATGAAT
GGCCTGATATTGAAGAAGATATTGCGTTGATCAAATCTGAAGAAGGAGAAAAAATGGTTTT
GGAGAATAACTTCTTCGTGGAAACCATGTTGCCATCAAAAATCATGAGAAAGTTAGAACCA
GAAGAATTTGCAGCATATCTTGAACCATTCAAAGAGAAAGGTGAAGTTCGTCGTCCAACATT
ATCATGGCCTCGTGAAATCCCGTTAGTAAAAGGTGGTAAACCTGACGTTGTACAAATTGTTA
GGAATTATAATGCTTATCTACGTGCAAGTGATGATTTACCAAAAATGTTTATTGAATCGGAC
CCAGGATTCTTTTCCAATGCTATTGTTGAAGGTGCCAAGAAGTTTCCTAATACTGAATTTGT
CAAAGTAAAAGGTCTTCATTTTTCGCAAGAAGATGCACCTGATGAAATGGGAAAATATATCA
AATCGTTCGTTGAGCGAGTTCTCAAAAATGAACAATAAGGCCGCGACTCTAGAGGGCCC

GUG-3XFLAG-RLuc (set 1)
TAATACGACTCACTATAGGGAGACCCAAGCTGGCTAGCGTTTAAACTTAAGCTTGGCAATC
CGGTACTGTTGGTAAATAAGCCACCGTGGACTACAAAGACCATGACGGTGATTATAAAGAT
CATGACATCGATTACAAGGATGACGATGACAAGACTTCGAAAGTTTATGATCCAGAACAAA
GGAAACGGATGATAACTGGTCCGCAGTGGTGGGCCAGATGTAAACAAATGAATGTTCTTGA
TTCATTTATTAATTATTATGATTCAGAAAAACATGCAGAAAATGCTGTTATTTTTTTACATGGT

AACGCGGCCTCTTCTTATTTATGGCGACATGTTGTGCCACATATTGAGCCAGTAGCGCGGT
GTATTATACCAGACCTTATTGGTATGGGCAAATCAGGCAAATCTGGTAATGGTTCTTATAGG
TTACTTGATCATTACAAATATCTTACTGCATGGTTTGAACTTCTTAATTTACCAAAGAAGATC
ATTTTTGTCGGCCATGATTGGGGTGCTTGTTTGGCATTTCATTATAGCTATGAGCATCAAGA
TAAGATCAAAGCAATAGTTCACGCTGAAAGTGTAGTAGATGTGATTGAATCATGGGATGAAT
GGCCTGATATTGAAGAAGATATTGCGTTGATCAAATCTGAAGAAGGAGAAAAAATGGTTTT
GGAGAATAACTTCTTCGTGGAAACCATGTTGCCATCAAAAATCATGAGAAAGTTAGAACCA
GAAGAATTTGCAGCATATCTTGAACCATTCAAAGAGAAAGGTGAAGTTCGTCGTCCAACATT
ATCATGGCCTCGTGAAATCCCGTTAGTAAAAGGTGGTAAACCTGACGTTGTACAAATTGTTA
GGAATTATAATGCTTATCTACGTGCAAGTGATGATTTACCAAAAATGTTTATTGAATCGGAC
CCAGGATTCTTTTCCAATGCTATTGTTGAAGGTGCCAAGAAGTTTCCTAATACTGAATTTGT
CAAAGTAAAAGGTCTTCATTTTTCGCAAGAAGATGCACCTGATGAAATGGGAAAATATATCA
AATCGTTCGTTGAGCGAGTTCTCAAAAATGAACAATAAGGCCGCGACTCTAGAGGGCCC

AAA-3XFLAG-RLuc (set 1)
TAATACGACTCACTATAGGGAGACCCAAGCTGGCTAGCGTTTAAACTTAAGCTTGGCAATC
CGGTACTGTTGGTAAATAAGCCACCAAAGACTACAAAGACCATGACGGTGATTATAAAGAT
CATGACATCGATTACAAGGATGACGATGACAAGACTTCGAAAGTTTATGATCCAGAACAAA
GGAAACGGATGATAACTGGTCCGCAGTGGTGGGCCAGATGTAAACAAATGAATGTTCTTGA
TTCATTTATTAATTATTATGATTCAGAAAAACATGCAGAAAATGCTGTTATTTTTTTACATGGT
AACGCGGCCTCTTCTTATTTATGGCGACATGTTGTGCCACATATTGAGCCAGTAGCGCGGT
GTATTATACCAGACCTTATTGGTATGGGCAAATCAGGCAAATCTGGTAATGGTTCTTATAGG
TTACTTGATCATTACAAATATCTTACTGCATGGTTTGAACTTCTTAATTTACCAAAGAAGATC
ATTTTTGTCGGCCATGATTGGGGTGCTTGTTTGGCATTTCATTATAGCTATGAGCATCAAGA
TAAGATCAAAGCAATAGTTCACGCTGAAAGTGTAGTAGATGTGATTGAATCATGGGATGAAT
GGCCTGATATTGAAGAAGATATTGCGTTGATCAAATCTGAAGAAGGAGAAAAAATGGTTTT
GGAGAATAACTTCTTCGTGGAAACCATGTTGCCATCAAAAATCATGAGAAAGTTAGAACCA
GAAGAATTTGCAGCATATCTTGAACCATTCAAAGAGAAAGGTGAAGTTCGTCGTCCAACATT
ATCATGGCCTCGTGAAATCCCGTTAGTAAAAGGTGGTAAACCTGACGTTGTACAAATTGTTA
GGAATTATAATGCTTATCTACGTGCAAGTGATGATTTACCAAAAATGTTTATTGAATCGGAC
CCAGGATTCTTTTCCAATGCTATTGTTGAAGGTGCCAAGAAGTTTCCTAATACTGAATTTGT
CAAAGTAAAAGGTCTTCATTTTTCGCAAGAAGATGCACCTGATGAAATGGGAAAATATATCA
AATCGTTCGTTGAGCGAGTTCTCAAAAATGAACAATAAGGCCGCGACTCTAGAGGGCCC

AUG-3XFLAG-RLuc (set 2)
TAATACGACTCACTATAGGGAGACCCAAGCTGGCTAGCGTTTAAACTTAAGCTTGGCAATC
CGGTACTGTTGGTAAATAAGCCCCCATGGACTACAAAGACCATGACGGTGATTATAAAGAT
CATGACATCGATTACAAGGATGACGATGACAAGACTTCGAAAGTTTATGATCCAGAACAAA
GGAAACGGATGATAACTGGTCCGCAGTGGTGGGCCAGATGTAAACAAATGAATGTTCTTGA
TTCATTTATTAATTATTATGATTCAGAAAAACATGCAGAAAATGCTGTTATTTTTTTACATGGT
AACGCGGCCTCTTCTTATTTATGGCGACATGTTGTGCCACATATTGAGCCAGTAGCGCGGT
GTATTATACCAGACCTTATTGGTATGGGCAAATCAGGCAAATCTGGTAATGGTTCTTATAGG
TTACTTGATCATTACAAATATCTTACTGCATGGTTTGAACTTCTTAATTTACCAAAGAAGATC
ATTTTTGTCGGCCATGATTGGGGTGCTTGTTTGGCATTTCATTATAGCTATGAGCATCAAGA
TAAGATCAAAGCAATAGTTCACGCTGAAAGTGTAGTAGATGTGATTGAATCATGGGATGAAT
GGCCTGATATTGAAGAAGATATTGCGTTGATCAAATCTGAAGAAGGAGAAAAAATGGTTTT
GGAGAATAACTTCTTCGTGGAAACCATGTTGCCATCAAAAATCATGAGAAAGTTAGAACCA
GAAGAATTTGCAGCATATCTTGAACCATTCAAAGAGAAAGGTGAAGTTCGTCGTCCAACATT
ATCATGGCCTCGTGAAATCCCGTTAGTAAAAGGTGGTAAACCTGACGTTGTACAAATTGTTA
GGAATTATAATGCTTATCTACGTGCAAGTGATGATTTACCAAAAATGTTTATTGAATCGGAC
CCAGGATTCTTTTCCAATGCTATTGTTGAAGGTGCCAAGAAGTTTCCTAATACTGAATTTGT

CAAAGTAAAAGGTCTTCATTTTTCGCAAGAAGATGCACCTGATGAAATGGAAAAATATATCA
AATCGTTCGTTGAGCGAGTTCTCAAAAATGAACAATTAAGGCCGCGACTCTAGAGGGCCC

CUG-3XFLAG-RLuc (set 2)
TAATACGACTCACTATAGGGAGACCCAAGCTGGCTAGCGTTTAAACTTAAGCTTGGCAATC
CGGTACTGTTGGTAAATAAGCCCCCCTGGACTACAAAGACCATGACGGTGATTATAAAGAT
CATGACATCGATTACAAGGATGACGATGACAAGACTTCGAAAGTTTATGATCCAGAACAAA
GGAAACGGATGATAACTGGTCCGCAGTGGTGGGCCAGATGTAAACAAATGAATGTTCTTGA
TTCATTTATTAATTATTATGATTCAGAAAAACATGCAGAAAATGCTGTTATTTTTTTACATGGT
AACGCGGCCTCTTCTTATTTATGGCGACATGTTGTGCCACATATTGAGCCAGTAGCGCGGT
GTATTATACCAGACCTTATTGGTATGGGCAAATCAGGCAAATCTGGTAATGGTTCTTATAGG
TTACTTGATCATTACAAATATCTTACTGCATGGTTTGAACTTCTTAATTTACCAAAGAAGATC
ATTTTTGTCGGCCATGATTGGGGTGCTTGTTTGGCATTTCATTATAGCTATGAGCATCAAGA
TAAGATCAAAGCAATAGTTCACGCTGAAAGTGTAGTAGATGTGATTGAATCATGGGATGAAT
GGCCTGATATTGAAGAAGATATTGCGTTGATCAAATCTGAAGAAGGAGAAAAAATGGTTTT
GGAGAATAACTTCTTCGTGGAAACCATGTTGCCATCAAAAATCATGAGAAAGTTAGAACCA
GAAGAATTTGCAGCATATCTTGAACCATTCAAAGAGAAAGGTGAAGTTCGTCGTCCAACATT
ATCATGGCCTCGTGAAATCCCGTTAGTAAAAGGTGGTAAACCTGACGTTGTACAAATTGTTA
GGAATTATAATGCTTATCTACGTGCAAGTGATGATTTACCAAAAATGTTTATTGAATCGGAC
CCAGGATTCTTTTCCAATGCTATTGTTGAAGGTGCCAAGAAGTTTCCTAATACTGAATTTGT
CAAAGTAAAAGGTCTTCATTTTTCGCAAGAAGATGCACCTGATGAAATGGAAAAATATATCA
AATCGTTCGTTGAGCGAGTTCTCAAAAATGAACAATTAAGGCCGCGACTCTAGAGGGCCC

GUG-3XFLAG-RLuc (set 2)
TAATACGACTCACTATAGGGAGACCCAAGCTGGCTAGCGTTTAAACTTAAGCTTGGCAATC
CGGTACTGTTGGTAAATAAGCCCCCGTGGACTACAAAGACCATGACGGTGATTATAAAGAT
CATGACATCGATTACAAGGATGACGATGACAAGACTTCGAAAGTTTATGATCCAGAACAAA
GGAAACGGATGATAACTGGTCCGCAGTGGTGGGCCAGATGTAAACAAATGAATGTTCTTGA
TTCATTTATTAATTATTATGATTCAGAAAAACATGCAGAAAATGCTGTTATTTTTTTACATGGT
AACGCGGCCTCTTCTTATTTATGGCGACATGTTGTGCCACATATTGAGCCAGTAGCGCGGT
GTATTATACCAGACCTTATTGGTATGGGCAAATCAGGCAAATCTGGTAATGGTTCTTATAGG
TTACTTGATCATTACAAATATCTTACTGCATGGTTTGAACTTCTTAATTTACCAAAGAAGATC
ATTTTTGTCGGCCATGATTGGGGTGCTTGTTTGGCATTTCATTATAGCTATGAGCATCAAGA
TAAGATCAAAGCAATAGTTCACGCTGAAAGTGTAGTAGATGTGATTGAATCATGGGATGAAT
GGCCTGATATTGAAGAAGATATTGCGTTGATCAAATCTGAAGAAGGAGAAAAAATGGTTTT
GGAGAATAACTTCTTCGTGGAAACCATGTTGCCATCAAAAATCATGAGAAAGTTAGAACCA
GAAGAATTTGCAGCATATCTTGAACCATTCAAAGAGAAAGGTGAAGTTCGTCGTCCAACATT
ATCATGGCCTCGTGAAATCCCGTTAGTAAAAGGTGGTAAACCTGACGTTGTACAAATTGTTA
GGAATTATAATGCTTATCTACGTGCAAGTGATGATTTACCAAAAATGTTTATTGAATCGGAC
CCAGGATTCTTTTCCAATGCTATTGTTGAAGGTGCCAAGAAGTTTCCTAATACTGAATTTGT
CAAAGTAAAAGGTCTTCATTTTTCGCAAGAAGATGCACCTGATGAAATGGAAAAATATATCA
AATCGTTCGTTGAGCGAGTTCTCAAAAATGAACAATTAAGGCCGCGACTCTAGAGGGCCC

AAA-3XFLAG-RLuc (set 2)
TAATACGACTCACTATAGGGAGACCCAAGCTGGCTAGCGTTTAAACTTAAGCTTGGCAATC
CGGTACTGTTGGTAAATAAGCCCCCAAAGACTACAAAGACCATGACGGTGATTATAAAGAT
CATGACATCGATTACAAGGATGACGATGACAAGACTTCGAAAGTTTATGATCCAGAACAAA
GGAAACGGATGATAACTGGTCCGCAGTGGTGGGCCAGATGTAAACAAATGAATGTTCTTGA
TTCATTTATTAATTATTATGATTCAGAAAAACATGCAGAAAATGCTGTTATTTTTTTACATGGT
AACGCGGCCTCTTCTTATTTATGGCGACATGTTGTGCCACATATTGAGCCAGTAGCGCGGT
GTATTATACCAGACCTTATTGGTATGGGCAAATCAGGCAAATCTGGTAATGGTTCTTATAGG

TTACTTGATCATTACAAATATCTTACTGCATGGTTTGAACTTCTTAATTTACCAAAGAAGATC
ATTTTTGTCGGCCATGATTGGGGTGCTTGTTTGGCATTTCATTATAGCTATGAGCATCAAGA
TAAGATCAAAGCAATAGTTCACGCTGAAAGTGTAGTAGATGTGATTGAATCATGGGATGAAT
GGCCTGATATTGAAGAAGATATTGCGTTGATCAAATCTGAAGAAGGAGAAAAAATGGTTTT
GGAGAATAACTTCTTCGTGGAAACCATGTTGCCATCAAAAATCATGAGAAAGTTAGAACCA
GAAGAATTTGCAGCATATCTTGAACCATTCAAAGAGAAAGGTGAAGTTCGTCGTCCAACATT
ATCATGGCCTCGTGAAATCCCGTTAGTAAAAGGTGGTAAACCTGACGTTGTACAAATTGTTA
GGAATTATAATGCTTATCTACGTGCAAGTGATGATTTACCAAAAATGTTTATTGAATCGGAC
CCAGGATTCTTTTCCAATGCTATTGTTGAAGGTGCCAAGAAGTTTCCTAATACTGAATTTGT
CAAAGTAAAAGGTCTTCATTTTTCGCAAGAAGATGCACCTGATGAAATGGAAAAATATATCA
AATCGTTCGTTGAGCGAGTTCTCAAAAATGAACAATAAGGCCGCGACTCTAGAGGGCCC

AUG-3XFLAG-RLuc (set 3)
TAATACGACTCACTATAGGGAGACCCAAGCTGGCTAGCGTTTAAACTTAAGCTTGGCAATC
CGGTACTGTTGGTAAATAAGCCCCCATGUGGGACTACAAAGACCATGACGGTGATTATAAA
GATCATGACATCGATTACAAGGATGACGATGACAAGACTTCGAAAGTTTATGATCCAGAAC
AAAGGAAACGGATGATAACTGGTCCGCAGTGGTGGGCCAGATGTAAACAAATGAATGTTCT
TGATTCATTTATTAATTATTATGATTCAGAAAAACATGCAGAAAATGCTGTTATTTTTTTACAT
GGTAACGCGGCCTCTTCTTATTTATGGCGACATGTTGTGCCACATATTGAGCCAGTAGCGC
GGTGTATTATACCAGACCTTATTGGTATGGGCAAATCAGGCAAATCTGGTAATGGTTCTTAT
AGGTTACTTGATCATTACAAATATCTTACTGCATGGTTTGAACTTCTTAATTTACCAAAGAAG
ATCATTTTTGTCGGCCATGATTGGGGTGCTTGTTTGGCATTTCATTATAGCTATGAGCATCA
AGATAAGATCAAAGCAATAGTTCACGCTGAAAGTGTAGTAGATGTGATTGAATCATGGGAT
GAATGGCCTGATATTGAAGAAGATATTGCGTTGATCAAATCTGAAGAAGGAGAAAAAATGG
TTTTGGAGAATAACTTCTTCGTGGAAACCATGTTGCCATCAAAAATCATGAGAAAGTTAGAA
CCAGAAGAATTTGCAGCATATCTTGAACCATTCAAAGAGAAAGGTGAAGTTCGTCGTCCAA
CATTATCATGGCCTCGTGAAATCCCGTTAGTAAAAGGTGGTAAACCTGACGTTGTACAAATT
GTTAGGAATTATAATGCTTATCTACGTGCAAGTGATGATTTACCAAAAATGTTTATTGAATCG
GACCCAGGATTCTTTTCCAATGCTATTGTTGAAGGTGCCAAGAAGTTTCCTAATACTGAATT
TGTCAAAGTAAAAGGTCTTCATTTTTCGCAAGAAGATGCACCTGATGAAATGGAAAAATATA
TCAAATCGTTCGTTGAGCGAGTTCTCAAAAATGAACAATAAGGCCGCGACTCTAGAGGGCC
C

CUG-3XFLAG-RLuc (set 3)
TAATACGACTCACTATAGGGAGACCCAAGCTGGCTAGCGTTTAAACTTAAGCTTGGCAATC
CGGTACTGTTGGTAAATAAGCCCCCCTGUGGGACTACAAAGACCATGACGGTGATTATAAA
GATCATGACATCGATTACAAGGATGACGATGACAAGACTTCGAAAGTTTATGATCCAGAAC
AAAGGAAACGGATGATAACTGGTCCGCAGTGGTGGGCCAGATGTAAACAAATGAATGTTCT
TGATTCATTTATTAATTATTATGATTCAGAAAAACATGCAGAAAATGCTGTTATTTTTTTACAT
GGTAACGCGGCCTCTTCTTATTTATGGCGACATGTTGTGCCACATATTGAGCCAGTAGCGC
GGTGTATTATACCAGACCTTATTGGTATGGGCAAATCAGGCAAATCTGGTAATGGTTCTTAT
AGGTTACTTGATCATTACAAATATCTTACTGCATGGTTTGAACTTCTTAATTTACCAAAGAAG
ATCATTTTTGTCGGCCATGATTGGGGTGCTTGTTTGGCATTTCATTATAGCTATGAGCATCA
AGATAAGATCAAAGCAATAGTTCACGCTGAAAGTGTAGTAGATGTGATTGAATCATGGGAT
GAATGGCCTGATATTGAAGAAGATATTGCGTTGATCAAATCTGAAGAAGGAGAAAAAATGG
TTTTGGAGAATAACTTCTTCGTGGAAACCATGTTGCCATCAAAAATCATGAGAAAGTTAGAA
CCAGAAGAATTTGCAGCATATCTTGAACCATTCAAAGAGAAAGGTGAAGTTCGTCGTCCAA
CATTATCATGGCCTCGTGAAATCCCGTTAGTAAAAGGTGGTAAACCTGACGTTGTACAAATT
GTTAGGAATTATAATGCTTATCTACGTGCAAGTGATGATTTACCAAAAATGTTTATTGAATCG
GACCCAGGATTCTTTTCCAATGCTATTGTTGAAGGTGCCAAGAAGTTTCCTAATACTGAATT
TGTCAAAGTAAAAGGTCTTCATTTTTCGCAAGAAGATGCACCTGATGAAATGGAAAAATATA

TCAAATCGTTCGTTGAGCGAGTTCTCAAAAATGAACAATAAGGCCGCGACTCTAGAGGGCC
C

GUG-3XFLAG-RLuc (set 3)
TAATACGACTCACTATAGGGAGACCCAAGCTGGCTAGCGTTTAAACTTAAGCTTGGCAATC
CGGTACTGTTGGTAAATAAGCCCCCGTGUGGGACTACAAAGACCATGACGGTGATTATAAA
GATCATGACATCGATTACAAGGATGACGATGACAAGACTTCGAAAGTTTATGATCCAGAAC
AAAGGAAACGGATGATAACTGGTCCGCAGTGGTGGGCCAGATGTAAACAAATGAATGTTCT
TGATTCATTTATTAATTATTATGATTCAGAAAAACATGCAGAAAATGCTGTTATTTTTTTACAT
GGTAACGCGGCCTCTTCTTATTTATGGCGACATGTTGTGCCACATATTGAGCCAGTAGCGC
GGTGTATTATACCAGACCTTATTGGTATGGGCAAATCAGGCAAATCTGGTAATGGTTCTTAT
AGGTTACTTGATCATTACAAATATCTTACTGCATGGTTTGAACTTCTTAATTTACCAAAGAAG
ATCATTTTTGTCGGCCATGATTGGGGTGCTTGTTTGGCATTTCATTATAGCTATGAGCATCA
AGATAAGATCAAAGCAATAGTTCACGCTGAAAGTGTAGTAGATGTGATTGAATCATGGGAT
GAATGGCCTGATATTGAAGAAGATATTGCGTTGATCAAATCTGAAGAAGGAGAAAAAATGG
TTTTGGAGAATAACTTCTTCGTGGAAACCATGTTGCCATCAAAAATCATGAGAAAGTTAGAA
CCAGAAGAATTTGCAGCATATCTTGAACCATTCAAAGAGAAAGGTGAAGTTCGTCGTCCAA
CATTATCATGGCCTCGTGAAATCCCGTTAGTAAAAGGTGGTAAACCTGACGTTGTACAAATT
GTTAGGAATTATAATGCTTATCTACGTGCAAGTGATGATTTACCAAAAATGTTTATTGAATCG
GACCCAGGATTCTTTTCCAATGCTATTGTTGAAGGTGCCAAGAAGTTTCCTAATACTGAATT
TGTCAAAGTAAAAGGTCTTCATTTTTCGCAAGAAGATGCACCTGATGAAATGGGAAAATATA
TCAAATCGTTCGTTGAGCGAGTTCTCAAAAATGAACAATAAGGCCGCGACTCTAGAGGGCC
C

AAA-3XFLAG-RLuc (set 3)
TAATACGACTCACTATAGGGAGACCCAAGCTGGCTAGCGTTTAAACTTAAGCTTGGCAATC
CGGTACTGTTGGTAAATAAGCCCCCAAAUGGGACTACAAAGACCATGACGGTGATTATAAA
GATCATGACATCGATTACAAGGATGACGATGACAAGACTTCGAAAGTTTATGATCCAGAAC
AAAGGAAACGGATGATAACTGGTCCGCAGTGGTGGGCCAGATGTAAACAAATGAATGTTCT
TGATTCATTTATTAATTATTATGATTCAGAAAAACATGCAGAAAATGCTGTTATTTTTTTACAT
GGTAACGCGGCCTCTTCTTATTTATGGCGACATGTTGTGCCACATATTGAGCCAGTAGCGC
GGTGTATTATACCAGACCTTATTGGTATGGGCAAATCAGGCAAATCTGGTAATGGTTCTTAT
AGGTTACTTGATCATTACAAATATCTTACTGCATGGTTTGAACTTCTTAATTTACCAAAGAAG
ATCATTTTTGTCGGCCATGATTGGGGTGCTTGTTTGGCATTTCATTATAGCTATGAGCATCA
AGATAAGATCAAAGCAATAGTTCACGCTGAAAGTGTAGTAGATGTGATTGAATCATGGGAT
GAATGGCCTGATATTGAAGAAGATATTGCGTTGATCAAATCTGAAGAAGGAGAAAAAATGG
TTTTGGAGAATAACTTCTTCGTGGAAACCATGTTGCCATCAAAAATCATGAGAAAGTTAGAA
CCAGAAGAATTTGCAGCATATCTTGAACCATTCAAAGAGAAAGGTGAAGTTCGTCGTCCAA
CATTATCATGGCCTCGTGAAATCCCGTTAGTAAAAGGTGGTAAACCTGACGTTGTACAAATT
GTTAGGAATTATAATGCTTATCTACGTGCAAGTGATGATTTACCAAAAATGTTTATTGAATCG
GACCCAGGATTCTTTTCCAATGCTATTGTTGAAGGTGCCAAGAAGTTTCCTAATACTGAATT
TGTCAAAGTAAAAGGTCTTCATTTTTCGCAAGAAGATGCACCTGATGAAATGGGAAAATATA
TCAAATCGTTCGTTGAGCGAGTTCTCAAAAATGAACAATAAGGCCGCGACTCTAGAGGGCC
C

HP+PV IRES-nLuc-3XFLAG
TAATACGACTCACTATAGGGCTCGAG**TTGGGGCGCGTGGTGGCGGCTGCAGCCGCCACC**
ACGCGCCCCGGAAAAAA**AGCTTGGGCTGCAGGTCTTAAAACAGCTCTGGGGTTGTACCC**
ACCCCAGAGGCCCACGTGGCGGCTAGTACTCCGGTATTGCGGTACCTTTGTACGCCTGT
TTTATACTCCCTTCCCCCGTAACTTAGAAGCACAATGTCCAAGTTCAATAGGAGGGGGTA
CAAACCAGTACCACCACGAACAAGCACTTCTGTTCCCCCGGTGAGGCTGTATAGGCTGT

TTCCACGGCTAAAAGCGGCTGATCCGTTATCCGCTCATGTACTTCGAGAAGCCTAGTATC ACCTTGGAATCTTCGATGCGTTGCGCTCAACACTCAACCCCAGAGTGTAGCTTAGGTCGA TGAGTCTGGACGTTCCTCACCGGCGACGGTGGTCCAGGCTGCGTTGGCGGCCTACCTGT GGCCCAAAGCCACAGGACGCTAGTTGTGAACAAGGTGTGAAGAGCCTATTGAGCTACCT GAGAGTCCTCCGGCCCCTGAATGCGGCTAATCCTAACCACGGAGCAGGCAGTGGCAAT CCAGCGACCAGCCTGTCGTAACGCGCAAGTTCGTGGCGGAACCGACTACTTTGGGTGTC CGTGTTTCCTTTTATTTTTACAATGGCTGCTTATGGTGACAATCATTGATTGTTATCATAAA GCAAATTGGATTGGCCATCCGGTGAGAATTTGATTATTAAATTACTCTCTTGTTGGGATTG CTCCTTTGAAATCTTGTGCACTCACACCTATTGGAATTACCTCATTGTTAAGATACAACAA CAACAACAACAACAACAACAACAACAACAACAACAACAACAACAACAACAACAACAACAACA ACAAATGGTCTTCACACTCGAAGATTTCGTTGGGGACTGGCGACAGACAGCCGGCTACAA CCTGGACCAAGTCCTTGAACAGGGAGGTGTGTCCAGTTTGTTTCAGAATCTCGGGGTGTC CGTAACTCCGATCCAAAGGATTGTCCTGAGCGGTGAAAATGGGCTGAAGATCGACATCCAT GTCATCATCCCGTATGAAGGTCTGAGCGGCGACCAAATGGGCCAGATCGAAAAAATTTTTA AGGTGGTGTACCCTGTGGATGATCATCACTTTAAGGTGATCCTGCACTATGGCACACTGGT AATCGACGGGGTTACGCCGAACATGATCGACTATTTCGGACGGCCGTATGAAGGCATCGC CGTGTTCGACGGCAAAAAGATCACTGTAACAGGGACCCTGTGGAACGGCAACAAAATTATC GACGAGCGCCTGATCAACCCCGACGGCTCCCTGCTGTTCCGAGTAACCATCAACGGAGTG ACCGGCTGGCGGCTGTGCGAACGCATTCTGGCGGACTACAAAGACCATGACGGTGATTAT AAAGATCATGACATCGATTACAAGGATGACGATGACAAGTAAGGCCGCGACTCTAGA

HP+PV IRES-(AUG to AAA)nLuc-3XFLAG
TAATACGACTCACTATAGGGCTCGAG**TTGGGGCGCGTGGTGGCGGCTGCAGCCGCCACC ACGCGCCCCGG**AAAAAA**AGCTTGGGCTGCAGGTCTTAAAACAGCTCTGGGGTTGTACCC ACCCCAGAGGCCCACGTGGCGGCTAGTACTCCGGTATTGCGGTACCTTTGTACGCCTGT TTTATACTCCCTTCCCCCGTAACTTAGAAGCACAATGTCCAAGTTCAATAGGAGGGGGTA CAAACCAGTACCACCACGAACAAGCACTTCTGTTCCCCCGGTGAGGCTGTATAGGCTGT TTCCACGGCTAAAAGCGGCTGATCCGTTATCCGCTCATGTACTTCGAGAAGCCTAGTATC ACCTTGGAATCTTCGATGCGTTGCGCTCAACACTCAACCCCAGAGTGTAGCTTAGGTCGA TGAGTCTGGACGTTCCTCACCGGCGACGGTGGTCCAGGCTGCGTTGGCGGCCTACCTGT GGCCCAAAGCCACAGGACGCTAGTTGTGAACAAGGTGTGAAGAGCCTATTGAGCTACCT GAGAGTCCTCCGGCCCCTGAATGCGGCTAATCCTAACCACGGAGCAGGCAGTGGCAAT CCAGCGACCAGCCTGTCGTAACGCGCAAGTTCGTGGCGGAACCGACTACTTTGGGTGTC CGTGTTTCCTTTTATTTTTACAATGGCTGCTTATGGTGACAATCATTGATTGTTATCATAAA GCAAATTGGATTGGCCATCCGGTGAGAATTTGATTATTAAATTACTCTCTTGTTGGGATTG CTCCTTTGAAATCTTGTGCACTCACACCTATTGGAATTACCTCATTGTTAAGATA**CAACAA CAACAACAACAACAACAACAACAACAACAACAACAACAACAACAACAACAACAACAACAACA ACAAAAAGTCTTCACACTCGAAGATTTCGTTGGGGACTGGCGACAGACAGCCGGCTACAA CCTGGACCAAGTCCTTGAACAGGGAGGTGTGTCCAGTTTGTTTCAGAATCTCGGGGTGTC CGTAACTCCGATCCAAAGGATTGTCCTGAGCGGTGAAAATGGGCTGAAGATCGACATCCAT GTCATCATCCCGTATGAAGGTCTGAGCGGCGACCAAATGGGCCAGATCGAAAAAATTTTTA AGGTGGTGTACCCTGTGGATGATCATCACTTTAAGGTGATCCTGCACTATGGCACACTGGT AATCGACGGGGTTACGCCGAACATGATCGACTATTTCGGACGGCCGTATGAAGGCATCGC CGTGTTCGACGGCAAAAAGATCACTGTAACAGGGACCCTGTGGAACGGCAACAAAATTATC GACGAGCGCCTGATCAACCCCGACGGCTCCCTGCTGTTCCGAGTAACCATCAACGGAGTG ACCGGCTGGCGGCTGTGCGAACGCATTCTGGCGGACTACAAAGACCATGACGGTGATTAT AAAGATCATGACATCGATTACAAGGATGACGATGACAAGTAAGGCCGCGACTCTAGA

HP+EMCV IRES-NLUC-3XFLAG
TAATACGACTCACTATAGGGAGACCCAAGCTGG**TTGGGGCGCGTGGTGGCGGCTGCAGC CGCCACCACGCGCCCCGG**CTAGTTAAGCTTGGTACCGAGCTCGGATCCGCCCTCGAGCG

GGATCAATTCCGCCCCCCCCCTAACGTTACTGGCCGAAGCCGCTTGGAATAAGGCCGGT
GTGCGTTTGTCTATATGTTATTTTCCACCATATTGCCGTCTTTTGGCAATGTGAGGGCCCG
GAAACCTGGCCCTGTCTTCTTGACGAGCATTCCTAGGGGTCTTTCCCCTCTCGCCAAAGG
AATGCAAGGTCTGTTGAATGTCGTGAAGGAAGCAGTTCCTCTGGAAGCTTCTTGAAGACA
AACAACGTCTGTAGCGACCCTTTGCAGGCAGCGGAACCCCCCACCTGGCGACAGGTGC
CTCTGCGGCCAAAAGCCACGTGTATAAGATACACCTGCAAAGGCGGCACAACCCCAGTG
CCACGTTGTGAGTTGGATAGTTGTGGAAAGAGTCAAATGGCTCTCCTCAAGCGTATTCAA
CAAGGGGCTGAAGGATGCCCAGAAGGTACCCCATTGTATGGGATCTGATCTGGGGCCTC
GGTGCACATGCTTTACATGTGTTTAGTCGAGGTTAAAAAACGTCTAGGCCCCCCGAACCA
CGGGGACGTGGTTTTCCTTTGAAAAACACGATGATAATATGGTCTTCACACTCGAAGATTT
CGTTGGGGACTGGCGACAGACAGCCGGCTACAACCTGGACCAAGTCCTTGAACAGGGAG
GTGTGTCCAGTTTGTTTCAGAATCTCGGGGTGTCCGTAACTCCGATCCAAAGGATTGTCCT
GAGCGGTGAAAATGGGCTGAAGATCGACATCCATGTCATCATCCCGTATGAAGGTCTGAG
CGGCGACCAAATGGGCCAGATCGAAAAAATTTTTAAGGTGGTGTACCCTGTGGATGATCAT
CACTTTAAGGTGATCCTGCACTATGGCACACTGGTAATCGACGGGGTTACGCCGAACATGA
TCGACTATTTCGGGCGGCCGTATGAAGGCATCGCCGTGTTCGACGGCAAAAAGATCACTG
TAACAGGGACCCTGTGGAACGGCAACAAAATTATCGACGAGCGCCTGATCAACCCCGACG
GCTCCCTGCTGTTCCGAGTAACCATCAACGGAGTGACCGGCTGGCGGCTGTGCGAACGC
ATTCTGGCGGACTACAAAGACCATGACGGTGATTATAAAGATCATGACATCGATTACAAGG
ATGACGATGACAAGTAAAAGGGTCAAGACAATTCTGCAGATATCCAGCACAGTGGCGGCC
GCTCGAGTCTAGA

HP+HCV IRES-NLUC-3XFLAG
TAATACGACTCACTATAGGGAGACCCAAGCTGGTTGGGGCGCGTGGTGGCGGCTGCAGC
CGCCACCACGCGCCCCGGCTAGTTAAGCTTGGTACCGAGCTCGGATCCCCTGTGAGGAA
CTACTGTCTTCACGCAGAAAGCGCCTAGCCATGGCGTTAGTATGAGTGTCGTACAGCCTC
CAGGCCCCCCCCTCCCGGGAGAGCCATAGTGGTCTGCGGAACCGGTGAGTACACCGGA
ATTGCCGGGAAGACTGGGTCCTTTCTTGGATAAACCCACTCTATGCCCGGCCATTTGGGC
GTGCCCCCGCAAGACTGCTAGCCGAGTAGCGTTGGGTTGCGAAAGGCCTTGTGGTACTG
CCTGATAGGGCGCTTGCGAGTGCCCCGGGAGGTCTCGTAGACCGTGCATCATGAGCAC
GAATCCTAAACCTCAAAGAAAAATGGTCTTCACACTCGAAGATTTCGTTGGGGACTGGCG
ACAGACAGCCGGCTACAACCTGGACCAAGTCCTTGAACAGGGAGGTGTGTCCAGTTTGTT
TCAGAATCTCGGGGTGTCCGTAACTCCGATCCAAAGGATTGTCCTGAGCGGTGAAAATGG
GCTGAAGATCGACATCCATGTCATCATCCCGTATGAAGGTCTGAGCGGCGACCAAATGGG
CCAGATCGAAAAAATTTTTAAGGTGGTGTACCCTGTGGATGATCATCACTTTAAGGTGATCC
TGCACTATGGCACACTGGTAATCGACGGGGTTACGCCGAACATGATCGACTATTTCGGAC
GGCCGTATGAAGGCATCGCCGTGTTCGACGGCAAAAAGATCACTGTAACAGGGACCCTGT
GGAACGGCAACAAAATTATCGACGAGCGCCTGATCAACCCCGACGGCTCCCTGCTGTTCC
GAGTAACCATCAACGGAGTGACCGGCTGGCGGCTGTGCGAACGCATTCTGGCGGACTAC
AAAGACCATGACGGTGATTATAAAGATCATGACATCGATTACAAGGATGACGATGACAAGT
AAAAGGGTCAAGACAATTCTGCAGATATCCAGCACAGTGGCGGCCGCTCGAGTCTAGAGG
GCCC

HP+CrPV IGR IRES-nLuc-3XFLAG
TAATACGACTCACTATAGGGAGACCCAAGCTGGTTGGGGCGCGTGGTGGCGGCTGCAGC
CGCCACCACGCGCCCCGGCTAGTTAAGCTTGGTACCGAGCTCGGATCCAGTACCCTTCAC
CAAAGCAAAAATGTGATCTTGCTTGTAAATACAATTTTGAGAGGTTAATAAATTACAAGTA
GTGCTATTTTTGTATTTAGGTTAGCTATTTAGCTTTACGTTCCAGGATGCCTAGTGGCAGC
CCCACAATATCCAGGAAGCCCTCTCTGCGGTTTTTCAGATTAGGTAGTCGAAAAACCTAA
GAAATTTACCTGCTACATTTCAAGATACCATGGTCTTCACACTCGAAGATTTCGTTGGGGA
CTGGCGACAGACAGCCGGCTACAACCTGGACCAAGTCCTTGAACAGGGAGGTGTGTCCA

GTTTGTTTCAGAATCTCGGGGTGTCCGTAACTCCGATCCAAAGGATTGTCCTGAGCGGTGA
AAATGGGCTGAAGATCGACATCCATGTCATCATCCCGTATGAAGGTCTGAGCGGCGACCA
AATGGGCCAGATCGAAAAAATTTTTAAGGTGGTGTACCCTGTGGATGATCATCACTTTAAG
GTGATCCTGCACTATGGCACACTGGTAATCGACGGGGTTACGCCGAACATGATCGACTATT
TCGGACGGCCGTATGAAGGCATCGCCGTGTTCGACGGCAAAAAGATCACTGTAACAGGGA
CCCTGTGGAACGGCAACAAAATTATCGACGAGCGCCTGATCAACCCCGACGGCTCCCTGC
TGTTCCGAGTAACCATCAACGGAGTGACCGGCTGGCGGCTGTGCGAACGCATTCTGGCG
GACTACAAAGACCATGACGGTGATTATAAAGATCATGACATCGATTACAAGGATGACGATG
ACAAGTAAAAGGGTCAAGACAATTCTGCAGATATCCAGCACAGTGGCGGCCGCTCGAGTC
TAGAGGGCCC

Recombinant protein sequences

MBP-tag
TEV protease site
His6-tag
mEGFP
eIF2A
FLAG-tag
Mxe GyrA Intein
Chitin Binding Domain (CBD)

His6-MBP DNA sequence

ATGGGTTCTTCTCACCATCACCATCACCATGGTTCTTCTATGAAAATCGAAGAAGGTAAACT
GGTAATCTGGATTAACGGCGATAAAGGCTATAACGGTCTCGCTGAAGTCGGTAAGAAATTC
GAGAAAGATACCGGAATTAAAGTCACCGTTGAGCATCCGGATAAACTGGAAGAGAAATTCC
CACAGGTTGCGGCAACTGGCGATGGCCCTGACATTATCTTCTGGGCACACGACCGCTTTG
GTGGCTACGCTCAATCTGGCCTGTTGGCTGAAATCACCCCGGACAAAGCGTTCCAGGACA
AGCTGTATCCGTTTACCTGGGATGCCGTACGTTACAACGGCAAGCTGATTGCTTACCCGAT
CGCTGTTGAAGCGTTATCGCTGATTTATAACAAAGATCTGCTGCCGAACCCGCCAAAAACC
TGGGAAGAGATCCCGGCGCTGGATAAAGAACTGAAAGCGAAAGGTAAGAGCGCGCTGAT
GTTCAACCTGCAAGAACCGTACTTCACCTGGCCGCTGATTGCTGCTGACGGGGGTTATGC
GTTCAAGTATGAAAACGGCAAGTACGACATTAAAGACGTGGGCGTGGATAACGCTGGCGC
GAAAGCGGGTCTGACCTTCCTGGTTGACCTGATTAAAAACAAACACATGAATGCAGACACC
GATTACTCCATCGCAGAAGCTGCCTTTAATAAAGGCGAAACAGCGATGACCATCAACGGCC
CGTGGGCATGGTCCAACATCGACACCAGCAAAGTGAATTATGGTGTAACGGTACTGCCGA
CCTTCAAGGGTCAACCATCCAAACCGTTCGTTGGCGTGCTGAGCGCAGGTATTAACGCCG
CCAGTCCGAACAAAGAGCTGGCAAAAGAGTTCCTCGAAAACTATCTGCTGACTGATGAAGG
TCTGGAAGCGGTTAATAAAGACAAACCGCTGGGTGCCGTAGCGCTGAAGTCTTACGAGGA
AGAGTTGGCGAAAGATCCACGTATTGCCGCCACTATGGAAAACGCCCAGAAAGGTGAAAT
CATGCCGAACATCCCGCAGATGTCCGCTTTCTGGTATGCCGTGCGTACTGCGGTGATCAA
CGCCGCCAGCGGTCGTCAGACTGTCGATGAAGCCCTGAAAGACGCGCAGACTAATGGGA
TCGAGGAAAACCTGTACTTCCAATCCAATATTGGAAGTGGATAA

His6-MBP protein sequence

MGSSHHHHHHGSSMKIEEGKLVIWINGDKGYNGLAEVGKKFEKDTGIKVTVEHPDKLEEKFPQ
VAATGDGPDIIFWAHDRFGGYAQSGLLAEITPDKAFQDKLYPFTWDAVRYNGKLIAYPIAVEALS
LIYNKDLLPNPPKTWEEIPALDKELKAKGKSALMFNLQEPYFTWPLIAADGGYAFKYENGKYDIK
DVGVDNAGAKAGLTFLVDLIKNKHMNADTDYSIAEAAFNKGETAMTINGPWAWSNIDTSKVNY
GVTVLPTFKGQPSKPFVGVLSAGINAASPNKELAKEFLENYLLTDEGLEAVNKDKPLGAVALKS
YEEELAKDPRIAATMENAQKGEIMPNIPQMSAFWYAVRTAVINAASGRQTVDEALKDAQTNGIE
ENLYFQSN

MBP-eIF2A-His6 DNA sequence

ATGGGTTCTTCTATGAAAATCGAAGAAGGTAAACTGGTAATCTGGATTAACGGCGATAAAG
GCTATAACGGTCTCGCTGAAGTCGGTAAGAAATTCGAGAAAGATACCGGAATTAAAGTCAC
CGTTGAGCATCCGGATAAACTGGAAGAGAAATTCCCACAGGTTGCGGCAACTGGCGATGG
CCCTGACATTATCTTCTGGGCACACGACCGCTTTGGTGGCTACGCTCAATCTGGCCTGTTG
GCTGAAATCACCCCGGACAAAGCGTTCCAGGACAAGCTGTATCCGTTTACCTGGGATGCC
GTACGTTACAACGGCAAGCTGATTGCTTACCCGATCGCTGTTGAAGCGTTATCGCTGATTT
ATAACAAAGATCTGCTGCCGAACCCGCCAAAAACCTGGGAAGAGATCCCGGCGCTGGATA
AAGAACTGAAAGCGAAAGGTAAGAGCGCGCTGATGTTCAACCTGCAAGAACCGTACTTCA
CCTGGCCGCTGATTGCTGCTGACGGGGGTTATGCGTTCAAGTATGAAAACGGCAAGTACG

ACATTAAAGACGTGGGCGTGGATAACGCTGGCGCGAAAGCGGGTCTGACCTTCCTGGTTG
ACCTGATTAAAAACAAACACATGAATGCAGACACCGATTACTCCATCGCAGAAGCTGCCTT
TAATAAAGGCGAAACAGCGATGACCATCAACGGCCCGTGGGCATGGTCCAACATCGACAC
CAGCAAAGTGAATTATGGTGTAACGGTACTGCCGACCTTCAAGGGTCAACCATCCAAACCG
TTCGTTGGCGTGCTGAGCGCAGGTATTAACGCCGCCAGTCCGAACAAAGAGCTGGCAAAA
GAGTTCCTCGAAAACTATCTGCTGACTGATGAAGGTCTGGAAGCGGTTAATAAAGACAAAC
CGCTGGGTGCCGTAGCGCTGAAGTCTTACGAGGAAGAGTTGGCGAAAGATCCACGTATTG
CCGCCACTATGGAAAACGCCCAGAAAGGTGAAATCATGCCGAACATCCCGCAGATGTCCG
CTTTCTGGTATGCCGTGCGTACTGCGGTGATCAACGCCGCCAGCGGTCGTCAGACTGTCG
ATGAAGCCCTGAAAGACGCGCAGACTAATGGGATCGAGGAAAACCTGTACTTCCAATCCAA
TGCAGCGCCGTCCACGCCGCTCTTGACAGTCCGAGGATCAGAAGGACTGTACATGGTGAA
TGGACCACCACATTTTACAGAAAGCACAGTGTTTCCAAGGGAATCTGGGAAGAATTGCAAA
GTCTGTATCTTTAGTAAGGATGGGACCTTGTTTGCCTGGGGCAATGGAGAAAAAGTAAATA
TTATCAGTGTCACTAACAAGGGACTACTGCACTCCTTCGACCTCCTGAAGGCAGTTTGCCT
TGAATTCTCACCCAAAAATACTGTCCTGGCAACGTGGCAGCCTTACACTACTTCTAAAGATG
GCACAGCTGGGATACCCAACCTACAACTTTATGATGTGAAAACTGGGACATGTTTGAAATC
TTTCATCCAGAAAAAAATGCAAAATTGGTGTCCATCCTGGTCAGAAGATGAAACTCTTTGTG
CCCGCAATGTTAACAATGAAGTTCACTTCTTTGAAAACAACAATTTTAACACAATTGCAAATA
AATTGCATTTGCAAAAAATTAATGATTTTGTATTATCACCTGGACCCCAACCATACAAGGTG
GCTGTCTATGTTCCAGGAAGTAAAGGTGCACCTTCATTTGTTAGATTATATCAGTACCCCAA
CTTTGCTGGACCTCATGCAGCTTTAGCTAATAAAAGTTTCTTTAAGGCAGATAAAGTTACAA
TGCTGTGGAATAAAAAAGCTACTGCTGTGTTGGTAATAGCTAGCACAGATGTTGACAAGAC
AGGAGCTTCCTACTATGGAGAACAAACTCTACACTACATTGCAACAAATGGAGAAAGTGCT
GTAGTGCAATTACCAAAAAATGGCCCCATTTATGATGTAGTTTGGAATTCTAGTTCTACTGA
GTTTTGTGCTGTATATGGTTTTATGCCTGCCAAAGCGACAATTTTCAACTTGAAATGTGATC
CTGTATTTGACTTTGGAACTGGTCCTCGTAATGCAGCCTACTATAGCCCTCATGGACATATA
TTAGTATTAGCTGGATTTGGAAATCTGAGGGGACAAATGGAAGTGTGGGATGTGAAAAACT
ACAAACTTATTTCTAAACCGGTGGCTTCTGATTCTACATATTTTGCTTGGTGCCCGGATGGT
GAGCATATTTTAACAGCTACATGTGCTCCCAGGTTACGGGTTAATAATGGATACAAAATTTG
GCATTATACTGGCTCTATCTTGCACAAGTATGATGTGCCATCAAATGCAGAATTATGGCAG
GTTTCTTGGCAGCCATTTTTGGATGGAATATTTCCAGCAAAAACAATAACTTACCAAGCAGT
TCCAAGTGAAGTACCCAATGAGGAACCTAAAGTTGCAACAGCTTATAGACCCCCAGCTTTA
AGAAATAAACCAATCACCAATTCCAAATTGCATGAAGAGGAACCACCTCAGAATATGAAACC
ACAATCAGGAAACGATAAGCCATTATCAAAAACAGCTCTTAAAAATCAAAGGAAGCATGAAG
CTAAGAAAGCTGCAAAGCAGGAAGCAAGAAGTGACAAGAGTCCAGATTTGGCACCTACTC
CTGCCCCACAGAGCACACCACGAAACACTGTCTCTCAGTCAATTTCTGGGGACCCTGAGAT
AGACAAAAAAATCAAGAACCTAAAGAAGAAACTGAAAGCAATCGAACAACTGAAAGAACAA
GCAGCAACTGGAAAACAGCTAGAAAAAAATCAGTTGGAGAAAATTCAGAAAGAAACAGCCC
TTCTCCAGGAGCTGGAAGATTTGGAATTGGGTATTGGTTCTTCTCACCATCACCATCACCAT
TAA

MBP-eIF2A-His6 protein sequence

MGSSMKIEEGKLVIWINGDKGYNGLAEVGKKFEKDTGIKVTVEHPDKLEEKFPQVAATGDGPDI
IFWAHDRFGGYAQSGLLAEITPDKAFQDKLYPFTWDAVRYNGKLIAYPIAVEALSLIYNKDLLPN
PPKTWEEIPALDKELKAKGKSALMFNLQEPYFTWPLIAADGGYAFKYENGKYDIKDVGVDNAG
AKAGLTFLVDLIKNKHMNADTDYSIAEAAFNKGETAMTINGPWAWSNIDTSKVNYGVTVLPTFK
GQPSKPFVGVLSAGINAASPNKELAKEFLENYLLTDEGLEAVNKDKPLGAVALKSYEEELAKDP
RIAATMENAQKGEIMPNIPQMSAFWYAVRTAVINAASGRQTVDEALKDAQTNGIEENLYFQSNA
APSTPLLTVRGSEGLYMVNGPPHFTESTVFPRESGKNCKVCIFSKDGTLFAWGNGEKVNIISVT
NKGLLHSFDLLKAVCLEFSPKNTVLATWQPYTTSKDGTAGIPNLQLYDVKTGTCLKSFIQKKMQ
NWCPSWSEDETLCARNVNNEVHFFENNNFNTIANKLHLQKINDFVLSPGPQPYKVAVYVPGSK

GAPSFVRLYQYPNFAGPHAALANKSFFKADKVTMLWNKKATAVLVIASTDVDKTGASYYGEQT
LHYIATNGESAVVQLPKNGPIYDVVWNSSSTEFCAVYGFMPAKATIFNLKCDPVFDFGTGPRNA
AYYSPHGHILVLAGFGNLRGQMEVWDVKNYKLISKPVASDSTYFAWCPDGEHILTATCAPRLR
VNNGYKIWHYTGSILHKYDVPSNAELWQVSWQPFLDGIFPAKTITYQAVPSEVPNEEPKVATAY
RPPALRNKPITNSKLHEEEPPQNMKPQSGNDKPLSKTALKNQRKHEAKKAAKQEARSDKSPDL
APTPAPQSTPRNTVSQSISGDPEIDKKIKNLKKKLKAIEQLKEQAATGKQLEKNQLEKIQKETALL
QELEDLELGIGSSHHHHHH

His6-mEGFP-FLAG DNA sequence

ATGGGTTCTTCTCACCATCACCATCACCATGGTTCTTCTGTGAGCAAGGGCGAGGAGCTGT
TCACCGGGGTGGTGCCCATCCTGGTCGAGCTGGACGGCGACGTAAACGGCCACAAGTTC
AGCGTGTCCGGCGAGGGCGAGGGCGATGCCACCTACGGCAAGCTGACCCTGAAGTTCAT
CTGCACCACCGGCAAGCTGCCCGTGCCCTGGCCCACCCTCGTGACCACCCTGACCTACG
GCGTGCAGTGCTTCAGCCGCTACCCCGACCACATGAAGCAGCACGACTTCTTCAAGTCCG
CCATGCCCGAAGGCTACGTCCAGGAGCGCACCATCTTCTTCAAGGACGACGGCAACTACA
AGACCCGCGCCGAGGTGAAGTTCGAGGGCGACACCCTGGTGAACCGCATCGAGCTGAAG
GGCATCGACTTCAAGGAGGACGGCAACATCCTGGGGCACAAGCTGGAGTACAACTACAAC
AGCCACAACGTCTATATCATGGCCGACAAGCAGAAGAACGGCATCAAGGTGAACTTCAAG
ATCCGCCACAACATCGAGGACGGCAGCGTGCAGCTCGCCGACCACTACCAGCAGAACAC
CCCCATCGGCGACGGCCCCGTGCTGCTGCCCGACAACCACTACCTGAGCACCCAGTCCA
AGCTGAGCAAAGACCCCAACGAGAAGCGCGATCACATGGTCCTGCTGGAGTTCGTGACCG
CCGCCGGGATCACTCTCGGCATGGACGAGCTGTACAAGGGTTCTTCTGATTATAAAGATGA
TGATGATAAAGTCTAA

His6-mEGFP-FLAG protein sequence

MGSSHHHHHHGSSVSKGEELFTGVVPILVELDGDVNGHKFSVSGEGEGDATYGKLTLKFICTT
GKLPVPWPTLVTTLTYGVQCFSRYPDHMKQHDFFKSAMPEGYVQERTIFFKDDGNYKTRAEV
KFEGDTLVNRIELKGIDFKEDGNILGHKLEYNYNSHNVYIMADKQKNGIKVNFKIRHNIEDGSVQ
LADHYQQNTPIGDGPVLLPDNHYLSTQSKLSKDPNEKRDHMVLLEFVTAAGITLGMDELYKGS
SDYKDDDDKV

MBP-eIF2A-His6-FLAG DNA sequence

ATGAAAATCGAAGAAGGTAAACTGGTAATCTGGATTAACGGCGATAAAGGCTATAACGGTC
TCGCTGAAGTCGGTAAGAAATTCGAGAAAGATACCGGAATTAAAGTCACCGTTGAGCATCC
GGATAAACTGGAAGAGAAATTCCCACAGGTTGCGGCAACTGGCGATGGCCCTGACATTAT
CTTCTGGGCACACGACCGCTTTGGTGGCTACGCTCAATCTGGCCTGTTGGCTGAAATCAC
CCCGGACAAAGCGTTCCAGGACAAGCTGTATCCGTTTACCTGGGATGCCGTACGTTACAA
CGGCAAGCTGATTGCTTACCCGATCGCTGTTGAAGCGTTATCGCTGATTTATAACAAAGAT
CTGCTGCCGAACCCGCCAAAAACCTGGGAAGAGATCCCGGCGCTGGATAAAGAACTGAAA
GCGAAAGGTAAGAGCGCGCTGATGTTCAACCTGCAAGAACCGTACTTCACCTGGCCGCTG
ATTGCTGCTGACGGGGGTTATGCGTTCAAGTATGAAAACGGCAAGTACGACATTAAAGACG
TGGGCGTGGATAACGCTGGCGCGAAAGCGGGTCTGACCTTCCTGGTTGACCTGATTAAAA
ACAAACACATGAATGCAGACACCGATTACTCCATCGCAGAAGCTGCCTTTAATAAAGGCGA
AACAGCGATGACCATCAACGGCCCGTGGGCATGGTCCAACATCGACACCAGCAAAGTGAA
TTATGGTGTAACGGTACTGCCGACCTTCAAGGGTCAACCATCCAAACCGTTCGTTGGCGTG
CTGAGCGCAGGTATTAACGCCGCCAGTCCGAACAAAGAGCTGGCAAAAGAGTTCCTCGAA
AACTATCTGCTGACTGATGAAGGTCTGGAAGCGGTTAATAAAGACAAACCGCTGGGTGCC
GTAGCGCTGAAGTCTTACGAGGAAGAGTTGGCGAAAGATCCACGTATTGCCGCCACTATG
GAAAACGCCCAGAAAGGTGAAATCATGCCGAACATCCCGCAGATGTCCGCTTTCTGGTAT
GCCGTGCGTACTGCGGTGATCAACGCCGCCAGCGGTCGTCAGACTGTCGATGAAGCCCT
GAAAGACGCGCAGACTAATGGGATCGAGGAAAACCTGTACTTCCAATCCAATGCAGCGCC

GTCCACGCCGCTCTTGACAGTCCGAGGATCAGAAGGACTGTACATGGTGAATGGACCACC
ACATTTTACAGAAAGCACAGTGTTTCCAAGGGAATCTGGGAAGAATTGCAAAGTCTGTATCT
TTAGTAAGGATGGGACCTTGTTTGCCTGGGGCAATGGAGAAAAAGTAAATATTATCAGTGT
CACTAACAAGGGACTACTGCACTCCTTCGACCTCCTGAAGGCAGTTTGCCTTGAATTCTCA
CCCAAAAATACTGTCCTGGCAACGTGGCAGCCTTACACTACTTCTAAAGATGGCACAGCTG
GGATACCCAACCTACAACTTTATGATGTGAAAACTGGGACATGTTTGAAATCTTTCATCCAG
AAAAAAATGCAAAATTGGTGTCCATCCTGGTCAGAAGATGAAACTCTTTGTGCCCGCAATG
TTAACAATGAAGTTCACTTCTTTGAAAACAACAATTTTAACACAATTGCAAATAAATTGCATT
TGCAAAAAATTAATGATTTTGTATTATCACCTGGACCCCAACCATACAAGGTGGCTGTCTAT
GTTCCAGGAAGTAAAGGTGCACCTTCATTTGTTAGATTATATCAGTACCCCAACTTTGCTGG
ACCTCATGCAGCTTTAGCTAATAAAAGTTTCTTTAAGGCAGATAAAGTTACAATGCTGTGGA
ATAAAAAAGCTACTGCTGTGTTGGTAATAGCTAGCACAGATGTTGACAAGACAGGAGCTTC
CTACTATGGAGAACAAACTCTACACTACATTGCAACAAATGGAGAAAGTGCTGTAGTGCAA
TTACCAAAAAATGGCCCCATTTATGATGTAGTTTGGAATTCTAGTTCTACTGAGTTTTGTGCT
GTATATGGTTTTATGCCTGCCAAAGCGACAATTTTCAACTTGAAATGTGATCCTGTATTTGA
CTTTGGAACTGGTCCTCGTAATGCAGCCTACTATAGCCCTCATGGACATATATTAGTATTAG
CTGGATTTGGAAATCTGAGGGGACAAATGGAAGTGTGGGATGTGAAAAACTACAAACTTAT
TTCTAAACCGGTGGCTTCTGATTCTACATATTTTGCTTGGTGCCCGGATGGTGAGCATATTT
TAACAGCTACATGTGCTCCCAGGTTACGGGTTAATAATGGATACAAAATTTGGCATTATACT
GGCTCTATCTTGCACAAGTATGATGTGCCATCAAATGCAGAATTATGGCAGGTTTCTTGGC
AGCCATTTTTGGATGGAATATTTCCAGCAAAAACAATAACTTACCAAGCAGTTCCAAGTGAA
GTACCCAATGAGGAACCTAAAGTTGCAACAGCTTATAGACCCCCAGCTTTAAGAAATAAAC
CAATCACCAATTCCAAATTGCATGAAGAGGAACCACCTCAGAATATGAAACCACAATCAGG
AAACGATAAGCCATTATCAAAAACAGCTCTTAAAAATCAAAGGAAGCATGAAGCTAAGAAAG
CTGCAAAGCAGGAAGCAAGAAGTGACAAGAGTCCAGATTTGGCACCTACTCCTGCCCCAC
AGAGCACACCACGAAACACTGTCTCTCAGTCAATTTCTGGGGACCCTGAGATAGACAAAAA
AATCAAGAACCTAAAGAAGAAACTGAAAGCAATCGAACAACTGAAAGAACAAGCAGCAACT
GGAAAACAGCTAGAAAAAAATCAGTTGGAGAAAATTCAGAAAGAAACAGCCCTTCTCCAGG
AGCTGGAAGATTTGGAATTGGGTATTGGTTCTTCTCACCATCACCATCACCATGGAGGATC
AGATTATAAAGATGATGATGATAAATAA

MBP-eIF2A-His6-FLAG protein sequence
MKIEEGKLVIWINGDKGYNGLAEVGKKFEKDTGIKVTVEHPDKLEEKFPQVAATGDGPDIIFWA
HDRFGGYAQSGLLAEITPDKAFQDKLYPFTWDAVRYNGKLIAYPIAVEALSLIYNKDLLPNPPKT
WEEIPALDKELKAKGKSALMFNLQEPYFTWPLIAADGGYAFKYENGKYDIKDVGVDNAGAKAG
LTFLVDLIKNKHMNADTDYSIAEAAFNKGETAMTINGPWAWSNIDTSKVNYGVTVLPTFKGQPS
KPFVGVLSAGINAASPNKELAKEFLENYLLTDEGLEAVNKDKPLGAVALKSYEEELAKDPRIAAT
MENAQKGEIMPNIPQMSAFWYAVRTAVINAASGRQTVDEALKDAQTNGIEENLYFQSNAAPST
PLLTVRGSEGLYMVNGPPHFTESTVFPRESGKNCKVCIFSKDGTLFAWGNGEKVNIISVTNKGL
LHSFDLLKAVCLEFSPKNTVLATWQPYTTSKDGTAGIPNLQLYDVKTGTCLKSFIQKKMQNWCP
SWSEDETLCARNVNNEVHFFENNNFNTIANKLHLQKINDFVLSPGPQPYKVAVYVPGSKGAPS
FVRLYQYPNFAGPHAALANKSFFKADKVTMLWNKKATAVLVIASTDVDKTGASYYGEQTLHYIA
TNGESAVVQLPKNGPIYDVVWNSSSTEFCAVYGFMPAKATIFNLKCDPVFDFGTGPRNAAYYS
PHGHILVLAGFGNLRGQMEVWDVKNYKLISKPVASDSTYFAWCPDGEHILTATCAPRLRVNNG
YKIWHYTGSILHKYDVPSNAELWQVSWQPFLDGIFPAKTITYQAVPSEVPNEEPKVATAYRPPA
LRNKPITNSKLHEEEPPQNMKPQSGNDKPLSKTALKNQRKHEAKKAAKQEARSDKSPDLAPTP
APQSTPRNTVSQSISGDPEIDKKIKNLKKKLKAIEQLKEQAATGKQLEKNQLEKIQKETALLQELE
DLELGIGSSHHHHHHGGSDYKDDDDK

eIF2A-FLAG protein sequence (from OriGene # TP304303)

MAPSTPLLTVRGSEGLYMVNGPPHFTESTVFPRESGKNCKVCIFSKDGTLFAWGNGEKVNIISV
TNKGLLHSFDLLKAVCLEFSPKNTVLATWQPYTTSKDGTAGIPNLQLYDVKTGTCLKSFIQKKM
QNWCPSWSEDETLCARNVNNEVHFFENNNFNTIANKLHLQKINDFVLSPGPQPYKVAVYVPGS
KGAPSFVRLYQYPNFAGPHAALANKSFFKADKVTMLWNKKATAVLVIASTDVDKTGASYYGEQ
TLHYIATNGESAVVQLPKNGPIYDVVWNSSSTEFCAVYGFMPAKATIFNLKCDPVFDFGTGPRN
AAYYSPHGHILVLAGFGNLRGQMEVWDVKNYKLISKPVASDSTYFAWCPDGEHILTATCAPRL
RVNNGYKIWHYTGSILHKYDVPSNAELWQVSWQPFLDGIFPAKTITYQAVPSEVPNEEPKVATA
YRPPALRNKPITNSKLHEEEPPQNMKPQSGNDKPLSKTALKNQRKHEAKKAAKQEARSDKSP
DLAPTPAPQSTPRNTVSQSISGDPEIDKKIKNLKKKLKAIEQLKEQAATGKQLEKNQLEKIQKETA
LLQELEDLKLGITRTRPLEQKLISEEDLAANDILDYKDDDDKV

eIF2A-Mxe GyrA Intein-CBD DNA sequence

ATGGCGCCGTCCACGCCGCTCTTGACAGTCCGAGGATCAGAAGGACTGTACATGGTGAAT
GGACCACCACATTTTACAGAAAGCACAGTGTTTCCAAGGGAATCTGGGAAGAATTGCAAAG
TCTGTATCTTTAGTAAGGATGGGACCTTGTTTGCCTGGGGCAATGGAGAAAAAGTAAATATT
ATCAGTGTCACTAACAAGGGACTACTGCACTCCTTCGACCTCCTGAAGGCAGTTTGCCTTG
AATTCTCACCCAAAAATACTGTCCTGGCAACGTGGCAGCCTTACACTACTTCTAAAGATGG
CACAGCTGGGATACCCAACCTACAACTTTATGATGTGAAAACTGGGACATGTTTGAAATCTT
TCATCCAGAAAAAAATGCAAAATTGGTGTCCATCCTGGTCAGAAGATGAAACTCTTTGTGCC
CGCAATGTTAACAATGAAGTTCACTTCTTTGAAAACAACAATTTTAACACAATTGCAAATAAA
TTGCATTTGCAAAAAATTAATGATTTTGTATTATCACCTGGACCCCAACCATACAAGGTGGC
TGTCTATGTTCCAGGAAGTAAAGGTGCACCTTCATTTGTTAGATTATATCAGTACCCCAACT
TTGCTGGACCTCATGCAGCTTTAGCTAATAAAAGTTTCTTTAAGGCAGATAAAGTTACAATG
CTGTGGAATAAAAAAGCTACTGCTGTGTTGGTAATAGCTAGCACAGATGTTGACAAGACAG
GAGCTTCCTACTATGGAGAACAAACTCTACACTACATTGCAACAAATGGAGAAAGTGCTGT
AGTGCAATTACCAAAAAATGGCCCCATTTATGATGTAGTTTGGAATTCTAGTTCTACTGAGT
TTTGTGCTGTATATGGTTTTATGCCTGCCAAAGCGACAATTTTCAACTTGAAATGTGATCCT
GTATTTGACTTTGGAACTGGTCCTCGTAATGCAGCCTACTATAGCCCTCATGGACATATATT
AGTATTAGCTGGATTTGGAAATCTGAGGGGACAAATGGAAGTGTGGGATGTGAAAAACTAC
AAACTTATTTCTAAACCGGTGGCTTCTGATTCTACATATTTTGCTTGGTGCCCGGATGGTGA
GCATATTTTAACAGCTACATGTGCTCCCAGGTTACGGGTTAATAATGGATACAAAATTTGGC
ATTATACTGGCTCTATCTTGCACAAGTATGATGTGCCATCAAATGCAGAATTATGGCAGGTT
TCTTGGCAGCCATTTTTGGATGGAATATTTCCAGCAAAAACAATAACTTACCAAGCAGTTCC
AAGTGAAGTACCCAATGAGGAACCTAAAGTTGCAACAGCTTATAGACCCCCAGCTTTAAGA
AATAAACCAATCACCAATTCCAAATTGCATGAAGAGGAACCACCTCAGAATATGAAACCACA
ATCAGGAAACGATAAGCCATTATCAAAAACAGCTCTTAAAAATCAAAGGAAGCATGAAGCTA
AGAAAGCTGCAAAGCAGGAAGCAAGAAGTGACAAGAGTCCAGATTTGGCACCTACTCCTG
CCCCACAGAGCACACCACGAAACACTGTCTCTCAGTCAATTTCTGGGGACCCTGAGATAGA
CAAAAAAATCAAGAACCTAAAGAAGAAACTGAAAGCAATCGAACAACTGAAAGAACAAGCA
GCAACTGGAAAACAGCTAGAAAAAAATCAGTTGGAGAAAATTCAGAAAGAAACAGCCCTTC
TCCAGGAGCTGGAAGATTTGGAATTGGGTATTTGCATCACGGGAGATGCACTAGTTGCCCT
ACCCGAGGGCGAGTCGGTACGCATCGCCGACATCGTGCCGGGTGCGCGGCCCAACAGTG
ACAACGCCATCGACCTGAAAGTCCTTGACCGGCATGGCAATCCCGTGCTCGCCGACCGGC
TGTTCCACTCCGGCGAGCATCCGGTGTACACGGTGCGTACGGTCGAAGGTCTGCGTGTGA
CGGGCACCGCGAACCACCCGTTGTTGTGTTTGGTCGACGTCGCCGGGGTGCCGACCCTG
CTGTGGAAGCTGATCGACGAAATCAAGCCGGGCGATTACGCGGTGATTCAACGCAGCGCA
TTCAGCGTCGACTGTGCAGGTTTTGCCCGCGGGAAACCCGAATTTGCGCCCACAACCTAC
ACAGTCGGCGTCCCTGGACTGGTGCGTTTCTTGGAAGCACACCACCGAGACCCGGACGC
CCAAGCTATCGCCGACGAGCTGACCGACGGGCGGTTCTACTACGCGAAAGTCGCCAGTGT
CACCGACGCCGGCGTGCAGCCGGTGTATAGCCTTCGTGTCGACACGGCAGACCACGCGT
TTATCACGAACGGGTTCGTCAGCCACGCTACTGGCCTCACCGGTCTGAACTCAGGCCTCA

CGACAAATCCTGGTGTATCCGCTTGGCAGGTCAACACAGCTTATACTGCGGGACAATTGGT
CACATATAACGGCAAGACGTATAAATGTTTGCAGCCCCACACCTCCTTGGCAGGATGGGAA
CCATCCAACGTTCCTGCCTTGTGGCAGCTTCAATAA

eIF2A-Mxe GyrA Intein-CBD protein sequence
MAPSTPLLTVRGSEGLYMVNGPPHFTESTVFPRESGKNCKVCIFSKDGTLFAWGNGEKVNIISV
TNKGLLHSFDLLKAVCLEFSPKNTVLATWQPYTTSKDGTAGIPNLQLYDVKTGTCLKSFIQKKM
QNWCPSWSEDETLCARNVNNEVHFFENNNFNTIANKLHLQKINDFVLSPGPQPYKVAVYVPGS
KGAPSFVRLYQYPNFAGPHAALANKSFFKADKVTMLWNKKATAVLVIASTDVDKTGASYYGEQ
TLHYIATNGESAVVQLPKNGPIYDVVWNSSSTEFCAVYGFMPAKATIFNLKCDPVFDFGTGPRN
AAYYSPHGHILVLAGFGNLRGQMEVWDVKNYKLISKPVASDSTYFAWCPDGEHILTATCAPRL
RVNNGYKIWHYTGSILHKYDVPSNAELWQVSWQPFLDGIFPAKTITYQAVPSEVPNEEPKVATA
YRPPALRNKPITNSKLHEEEPPQNMKPQSGNDKPLSKTALKNQRKHEAKKAAKQEARSDKSP
DLAPTPAPQSTPRNTVSQSISGDPEIDKKIKNLKKKLKAIEQLKEQAATGKQLEKNQLEKIQKETA
LLQELEDLELGICITGDALVALPEGESVRIADIVPGARPNSDNAIDLKVLDRHGNPVLADRLFHSG
EHPVYTVRTVEGLRVTGTANHPLLCLVDVAGVPTLLWKLIDEIKPGDYAVIQRSAFSVDCAGFA
RGKPEFAPTTYTVGVPGLVRFLEAHHRDPDAQAIADELTDGRFYYAKVASVTDAGVQPVYSLR
VDTADHAFITNGFVSHATGLTGLNSGLTTNPGVSAWQVNTAYTAGQLVTYNGKTYKCLQPHTS
LAGWEPSNVPALWQLQ
